# Cation redistribution enables low-temperature stabilization of MA-free FAPbI_3_

**DOI:** 10.1039/d6el00108d

**Published:** 2026-09-30

**Authors:** Riccardo Pallotta, Simone Filippi, Alice Scardina, Weidong Xu, Samuele Mattioni, Mingjie Feng, Sergio Marras, Ruggero Sala, Matteo Degani, Luis E. Hueso, Tianyu Xu, Beatriz Martín-García, Chang-Qi Ma, Andres Osvet, Samuel D. Stranks, Christoph J. Brabec, Giulia Grancini

**Affiliations:** a Department of Chemistry, INSTM, University of Pavia Via T. Taramelli 1,4 Pavia 27100 Italy giulia.grancini@unipv.it; b Institute of Materials for Electronics and Energy Technology (i-MEET), Department of Materials Science and Engineering, Friedrich-Alexander-Universität Erlangen-Nürnberg Martensstraße 7 Erlangen 91058 Germany; c Department of Chemical Engineering & Biotechnology, University of Cambridge Philippa Fawcett Drive Cambridge CB3 0AS UK; d Department of Physics, Cavendish Laboratory, University of Cambridge JJ Thomson Avenue Cambridge CB3 0HE UK; e CIC nanoGUNE BRTA Tolosa Hiribidea, 76 Donostia-San Sebastián 20018 Basque Country Spain; f Departamento de Polímeros y Materiales Avanzados: Física, Química y Tecnología, University of the Basque Country (UPV/EHU) Donostia-San Sebastián 20018 Basque Country Spain; g Materials Characterization Facility, Istituto Italiano di Tecnologia Via Morego 16163 Genova Italy; h IKERBASQUE-Basque Foundation for Science Bilbao 48009 Basque Country Spain; i i-Lab & Printable Electronics Research Center, Suzhou Institute of Nano-Tech and Nano-Bionics (SINANO), Chinese Academy of Sciences (CAS) Ruoshui Road 398 Suzhou 215123 China; j Helmholtz-Institute Erlangen-Nürnberg for Renewable Energy (HI ERN) Immerwahrstraße 2 Erlangen 91058 Germany

## Abstract

Metal halide perovskites have reshaped photovoltaic research, with formamidinium lead triiodide (FAPbI_3_) standing out as a top contender for single-junction solar cells due to its ideal band gap. However, obtaining its photoactive black phase, especially at low processing temperatures, remains a key challenge, as it tends to degrade into the undesirable yellow δ-phase. While conventional strategies to stabilize the cubic FAPbI_3_ phase rely on methylammonium (MA)-assisted formulations, which can compromise long-term stability and inevitably increase the band gap, we present an MA-free approach that combines a small amount of cesium (2%) with a binary antisolvent system. The addition of a polar alcoholic component promotes FA^+^ redistribution during film formation, enabling uniform incorporation of the limited Cs^+^ content throughout the perovskite layer. As a result, the α-phase is stabilized at 100 °C, while maintaining a narrow band gap close to 1.5 eV, demonstrating a promising route toward the low-temperature processing of FA-based photovoltaic devices.

## Abstract 2

IPA facilitates FA^+^ redistribution, thereby promoting a more homogeneous distribution of Cs^+^ throughout the film and, consequently, enabling uniform and complete conversion to the α-phase.

## Graphical abstract

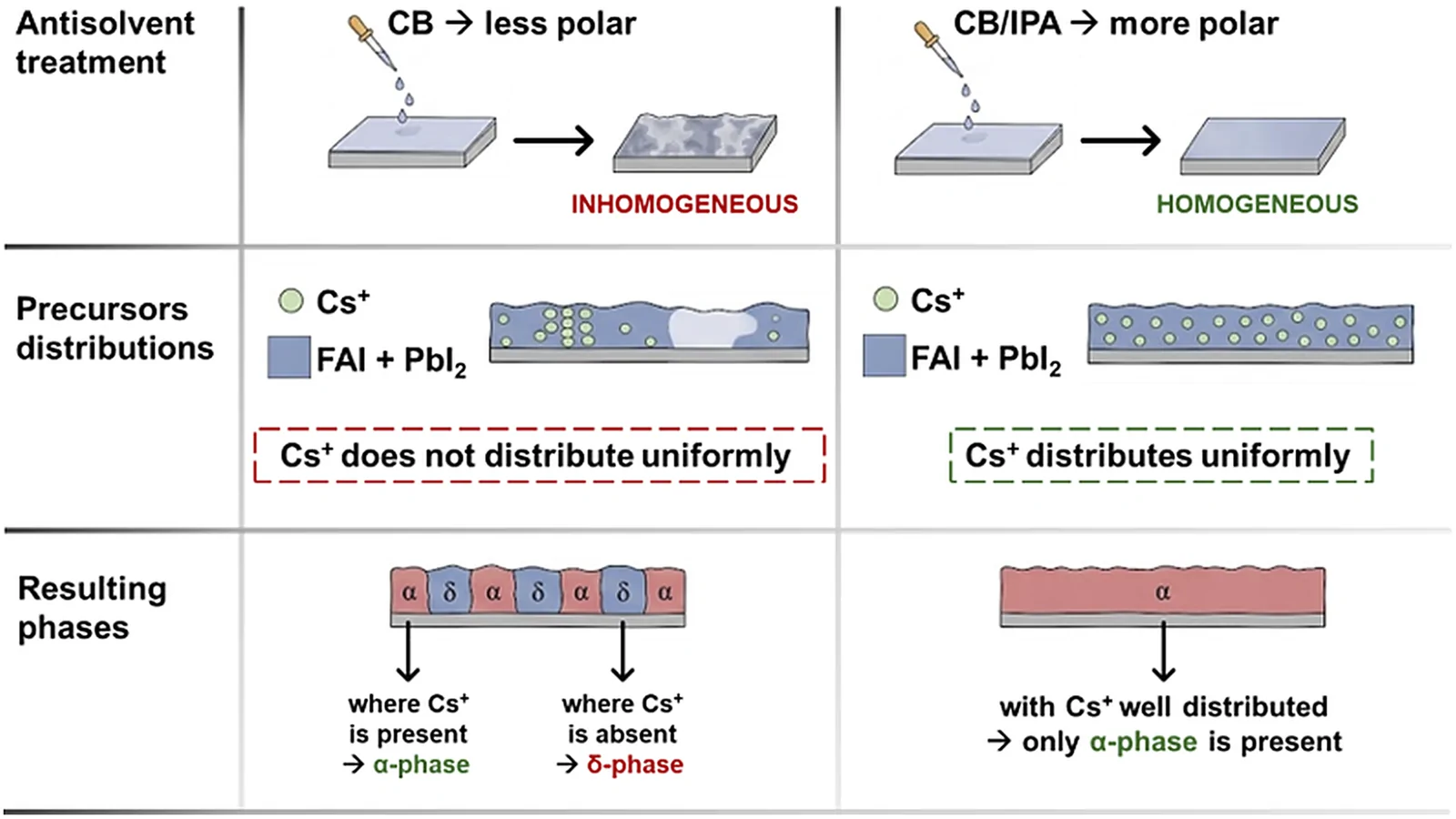

**Broader context**

Within metal halide perovskites, FAPbI_3_ is a promising absorber for single-junction solar cells due to its narrow band gap, but its practical use is limited by the instability of the photoactive black phase at low processing temperatures. Conventional stabilization strategies often rely on methylammonium incorporation, which undesirably widens the band gap. In this work, a MA-free strategy is presented based on the introduction of a small amount of cesium (2%) together with an optimized binary antisolvent system. This combination enables controlled crystallization and a more homogeneous Cs distribution throughout the film thickness, leading to stabilization of the black phase at low processing temperatures. As a result, a band gap close to 1.5 eV is maintained while processing is carried out at only 100 °C.

## Introduction

Research on Metal Halide Perovskites (MHPs) has recently approached the commercial scale in tandem configurations.^1–3^ While a wide range of material band gaps have been explored to adapt to different technologies,^4–7^ low band gap materials are the most promising for single-junction solar cell devices.^8^ Several studies have shown that the realization of narrow band gap perovskite devices is crucial for achieving state-of-the-art photovoltaic performance, and recent progress with methylammonium (MA)-free formamidinium lead triiodide (FAPbI_3_) absorbers has delivered record efficiencies while ensuring improved stability.^9,10^ In this context, FAPbI_3_ is an ideal solution due to its suitable bandgap of around 1.5 eV.^8,11,12^ Currently, the main challenge when working with pure FA-based MHPs is the high temperature (∼150 °C) required for the phase transition to occur from the optically inactive non-perovskite δ-phase (yellow phase) to the optically active α-phase (black phase) and the poor quality of the layer obtained.^13,14^ This issue has been mainly addressed by introducing MA-based additives (*e.g.*, methylammonium chloride, MACl) and by multi cation compositional engineering incorporating cesium (Cs^+^) and MA^+^ and traces of bromide (Br^−^).^15–19^ On the one hand, this strategy successfully reduces the annealing temperature of the perovskite active layer to around 100 °C. On the other hand, the inclusion of smaller cations and halides, typically in concentrations ranging from 5% to 15% mol mol^−1^ relative to the perovskite, inevitably widens the band gap and introduces phase segregation.^20–22^ Only a few studies have reported methods to stabilize α-FAPbI_3_ at 100 °C without using MA-based additives. For instance, Tiarnan *et al.* achieved this stabilization by inducing octahedral tilting, which promotes the phase transition from the cubic to the tetragonal phase, through the incorporation of acetic acid-based additives into the perovskite precursor solution.^23^ Li *et al.*, instead, obtained a stable MA-free FAPbI_3_ by incorporating triphenylphosphine oxide at the interface between the perovskite and the electron transport layer.^24^ Despite their effectiveness, conventional approaches rely on extrinsic stabilization strategies based on the incorporation of external additives or complex compositions; here, we propose an alternative stabilization strategy that enables the formation of the cubic α-phase of FAPbI_3_ without MA-based additives and using a low Cs^+^ content. Specifically, we employ only 2% Cs^+^ in the precursor solution, substantially lower than the concentrations commonly used in mixed-cation formulations, in combination with a polar alcoholic component introduced into the antisolvent system. Importantly, the use of the low Cs^+^ content alone was not sufficient to achieve complete and homogeneous α-phase conversion at low temperature, highlighting the synergistic role of the two components. The polar alcoholic component was found to favour the redistribution of FA^+^ during film formation, which in turn promotes a homogeneous distribution of the small amount of Cs^+^ throughout the perovskite layer. This combined effect enables a more uniform conversion of the active material from the δ- to the α-FAPbI_3_ phase at low temperature, as demonstrated by Time-of-Flight Secondary Ion Mass Spectrometry (ToF-SIMS) and photoluminescence (PL) mapping. Refinement of the XRD patterns revealed that FAPbI_3_ processed using this method exhibits larger lattice parameters than FAPbI_3_ prepared using conventional MACl additives, resulting in a reduced band gap. We evaluated our MA-free FAPbI_3_ in solar cell devices with an inverted (p–i–n) architecture, using FAPbI_3_ films processed with either the binary solvent system or individual solvents. While devices prepared using individual solvents exhibited low power conversion efficiencies (PCEs), mainly due to incomplete conversion of the active layer, those fabricated using the binary solvent system achieved PCEs approaching 22%, confirming the effectiveness of our strategy for the fabrication of MA-free FAPbI_3_ solar cells.

## Experimental

### Materials

Chlorobenzene (CB, extra dry, 99.8%), dimethyl sulfoxide (DMSO, ≥99.9% extra dry), *N*,*N*-dimethylformamide (DMF, extra dry, 99.8%) and chloroform (CF, extra dry, 99.8%) were purchased from Acros Organics. 2-Propanol (IPA, ≥99.8%), lead iodide (PbI_2_, >98.0%) and MeO-2PACz were purchased from TCI. Formamidinium iodide (FAI, >99.99%), cesium iodide (CsI, >99.99%) and methylammonium chloride (MACl, >99.99%) were purchased from Greatcell. Phenyl-C_61_-butyric acid methyl ester (PCBM, >99.99%) was purchased from Lumatec. Ethanol (extra dry, 99.8%) and bathocuproine (BCP) were purchased from Sigma-Aldrich. All solutions were prepared in an Ar-filled glovebox, while the deposition of each layer of the solar cell was performed in an N_2_-filled glovebox.

### Device fabrication

For the fabrication of perovskite solar cells (PSCs), indium tin oxide (ITO)-coated glass substrates (purchased from Yingkou Shangneng Photoelectric Material Co., Ltd) were consecutively cleaned in acetone and IPA by ultrasonication for 15 min for each solvent. The substrates were dried with N_2_ airflow and O_2_ plasma treated for 10 min. MeO-2PACz was dissolved in ethanol and 50 µL were spin-coated onto ITO/glass substrates at 3000 rpm for 30 s and annealed at 100 °C for 10 min. The MA-free perovskite precursor solution was prepared by dissolving 0.726 g of PbI_2_, 0.253 g of FAI and 7.8 mg of CsI (for 2% samples) powders in 1 mL DMF/DMSO 4/1 vol/vol solution. For MACl-FAPbI_3_, 25% mol mol^−1^ of MACl was added to the precursor solution. 25 µL of the final solution were deposited on the MeO-2PACz coated substrates and spin-coated with a three-step procedure: the first step proceeded at 1000 rpm (500 rpm per s) for 6 s and the second step proceeded at 5000 rpm for 27 s, while the last step was a speed reduction of 1250 rpm per s. 200 µL of CB, CB/IPA 2/1 v/v, and IPA were added dropwise onto the spinning substrate for an antisolvent procedure after 20 s from the beginning of the second step. Subsequently, substrates were annealed at 100 °C for 30 min. To fabricate the ETL, PCBM was dissolved in chloroform to produce a 15 mg mL^−1^ solution. 20 µL of the solution were spin-coated at 2000 rpm for 20 s onto the perovskite layer. To prevent the diffusion of the metal contact inside the perovskite, 50 µL of 1 mg mL^−1^ solution (in isopropanol) of bathocuproine were deposited on PCBM. Finally, 80 nm of Ag was thermally evaporated onto the device through a shadow mask with an area of 0.0825 cm^2^. The evaporation speed was adjusted to 0.01 nm s^−1^ for the first 5 nm, 0.02 nm s^−1^ from 5 to 15 nm, and 0.06 nm s^−1^ for the rest of the procedure. A vacuum-based chuck was used for all depositions.

### Device characterization

The current density–voltage (*J*–*V*) characteristics of the produced PSCs were determined using a Keithley 2400 source meter and an Abet solar simulator. The simulated 1 Sun AM1.5G illumination was calibrated using a certified Si reference cell (Open RR-1002, KG5 window). The devices were measured in both reverse scan (1.2 V → 0 V, 130 mV s^−1^) and forward scan (0 → 1.2 V, 130 mV s^−1^). During every measurement, a metal shadow mask with an aperture area of 0.03 cm^2^ was used to cover the device's active area. Incident Photon to Current Conversion Efficiency (IPCE) was determined using the Cicci Research Arkeo transient tests module. Measurements were conducted under 1 Sun illumination and constant bias was perturbed with short light pulses of 200 µs width.

### Maximum power point tracking

The stability measurements were performed on unencapsulated devices at the maximum power point (according to the ISOS-L-1 protocol) under nitrogen using a stability box (Litos Lite) from Fluxim, coupled with a Wavelabs solar simulator continuously illuminating the devices. The spectrum used for stability measurements was the same as the one employed for current density voltage measurements.

### Statistical analysis

For photovoltaic parameters statistical analysis, data were pre-processed by defining as outliers all values lying above or below the 1.5 interquartile range. The resulting dataset was presented as box charts, where the upper and lower limits of the box identify the 75th and 25th percentile, respectively, while whiskers indicate 1.5 times the interquartile range. The mean value for each sample is presented as a horizontal line within the box. OriginPro 2018 was employed for statistical analysis.

### Morphology and structural characterization

Top view scanning electron microscopy (SEM) images of the films at different magnifications were collected using a FEI® Helios Nanolab 450S instrument using 5 kV and 0.2 nA as conditions for imaging. The same accelerating voltage and working distance were used for all the samples. X-ray diffraction analysis was carried out on a Malvern-PANalytical 3rd generation Empyrean X-ray powder diffractometer equipped with a 1.8 kW CuKα ceramic X-ray tube operating at 45 kV and 40 mA, iCore and dCore automated PreFIX optical modules, a motorized Eulerian Cradle (chi, phi, *x*, *y* and *z* movements) and a solid-state hybrid pixel PIXcel3D detector operating in 1D mode. The XRD patterns were collected in Bragg–Brentano geometry from 10° to 65° with a step size of 0.013° in automatic variable slit mode. Data analysis was performed with HighScore Plus 5.3a.

### Optical analysis

UV-Vis absorption spectra of the perovskite thin films were collected using a commercial UV-NIR spectrophotometer (Lambda1050+, PerkinElmer).

Wide-field, hyperspectral microscopy measurements were conducted using a Photon *etc.* IMA system. An Olympus NA = 0.9 objective lens, with 100× magnification, was used for all measurements. To correct for aberrations stemming from the optical elements in the system, the apparatus was calibrated using a reference sample to determine the post-processing parameters that were used to correct the image distortion. Chromatic aberrations were mitigated by calibrating the *z* position of the sample for every collected wavelength. To minimize oxygen and humidity-related degradation behavior, samples with the structure of quartz/perovskite were stored in a nitrogen-filled glovebox immediately prior to measurement. A 405 nm continuous wave laser served as the excitation source. The excitation laser was filtered by a high-quality 405 nm Semrock dichroic mirror to separate the photoluminescence and excitation signals. The emitted light from the sample was incident on a volume Bragg grating, which spectrally split the light onto a silicon Hamamatsu CMOS camera with an array of 2048 × 2048 pixels. A two-step calibration process was employed for each objective lens to extract the absolute number of photons at each point. First, a calibrated white light lamp was coupled to an integrating sphere, through the objective lens. The measured lamp spectrum at each point was compared to the lamp's known spectrum, establishing the system's relative sensitivity both spectrally and spatially. Subsequently, a 657 nm laser with a known optical power was fiber coupled and imaged using the microscope. Hyperspectral measurement of the fiber output enabled the conversion between the number of counts and photons at this wavelength. Combining this absolute calibration with the relative calibration from the calibrated white light lamp and integrating sphere made it possible to accurately quantify the number of photons emitted across the spectrum at each point of the sample. The spectral centre of mass for each point was calculated by interpolating the data along a uniform energy axis (*E*_*n*_ = *hc*/*λ*) and then calculated as
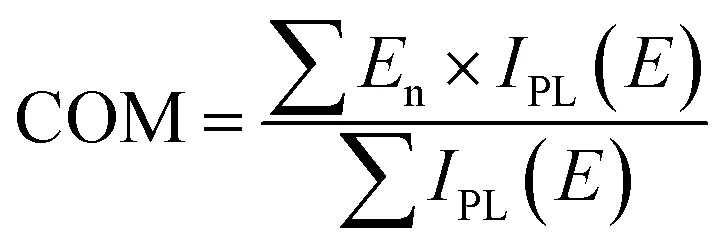
where *I*_PL_ (*E*) is the absolute intensity of photoluminescence, ℎ is Planck's constant, *c* is the speed of light and *λ* is the wavelength.^25^

## Results and discussion

It is widely accepted that the chemical and physical properties of solvents can have a strong impact on perovskite crystallization.^26–29^ In our study, we explored the effect of a binary solvent system introducing isopropanol (IPA) as a second component in the antisolvent, along with the commonly used chlorobenzene (CB),^30^ aiming at investigating its impact on FAPbI_3_ crystallization. Due to the low polarity of IPA, we were able to avoid damage to the perovskite layer during the antisolvent treatment, as recently demonstrated.^29,31,32^ First, to study the effects of solvents on FAPbI_3_ conversion (at 100 °C), we deposited a matrix of FAPbI_3_ thin films by simultaneously changing the CB/IPA ratio and the percentage of Cs^+^. To remain as close as possible to pristine FAPbI_3_ and minimize band-gap widening associated with the incorporation of additional A-site cations, we investigated the minimum Cs^+^ content required to stabilize the α-FAPbI_3_ phase at 100 °C by progressively increasing its concentration from 0% to 1% and 2%.

Fig. 1(a) and S1 report X-ray Diffraction (XRD) patterns of FAPbI_3_ thin films with different percentages of Cs^+^ processed with single (CB and IPA) and blended solvents in different ratios, 1/1 v/v, 2/1 v/v and 4/1 v/v (named CB/IPA1, CB/IPA2 and CB/IPA4, respectively). With 0% Cs^+^ the XRD patterns, independently of the solvent, are dominated by the presence of a peak at 2*θ* = 11.6° related to the hexagonal non-perovskite δ-phase (Fig. S1a).^33^ However, by increasing the concentration of Cs^+^ to 1%, in the CB/IPA1 and CB/IPA2 samples, the characteristic diffraction peaks related to the FAPbI_3_ α-phase at 2*θ* = 14.0° started to appear (Fig. S1b).^34^ This trend continued by additionally increasing the concentration of Cs^+^ to 2%. Fig. 1(a) shows, for the CB/IPA2-2%, the presence of sharp peaks at 2*θ* = 14.0° and 2*θ* = 19.7° related to the (100) and (110) lattice planes of the FAPbI_3_ black phase, respectively. Since no MACl was included, we exclude that the stabilization effect is related to the formation of an isopropylammonium chloride intermediate given by the reaction between IPA and MACl, as recently reported.^35^ Note that the peak related to lead iodide (PbI_2_) at 2*θ* = 12.6° is due to the presence of an excess voluntarily added in the precursor solution.^36–38^ Interestingly, in the IPA-2% and CB-2% samples, a large amount of δ-phase was still present, while CB/IPA1-2% and CB/IPA4-2% represented an intermediate step (Fig. S1c). In Fig. S2 the phases present in all the different thin films processed with different solvents are schematized. In Fig. S3a–f pictures of thin films with different percentages of Cs^+^ processed with various solvents are reported. To further elucidate the effect of the solvents on FAPbI_3_ conversion, we studied the Full Width at Half Maximum (FWHM) of the peak related to the (100) crystallographic plane of CB-2%, CB/IPA2-2% and IPA-2% samples (Fig. 1(b)). By analyzing the FWHM, we can extract information about the crystallite size, according to the Scherrer equation.^39^ In particular, the lower FWHM (Fig. 1(c)) for the CB/IPA2-2% with respect to the CB-2% and IPA-2% samples indicated a greater crystallite size, confirming the positive effect of solvent blend in facilitating the formation of the FAPbI_3_ black phase. Besides structural information, the thin film morphology is also a key aspect in determining semiconductor quality.^40,41^ To this end, we performed Scanning Electron Microscopy (SEM) analysis on CB-2%, CB/IPA2-2% and IPA-2%; results are reported inFig. 1(d)–(f). From the SEM images, we observed poorly crystalline and highly agglomerated small grains for the CB-2% and IPA-2%. On the other hand, CB/IPA2-2% showed distinct and uniform grains along the thin film surface. These results are consistent with the mixed phases and broader peaks observed in the XRD patterns of the samples treated with single solvents. This consideration is even clearer when looking at SEM images at different magnifications (Fig. S4a–i). Indeed, the different color contrast that emerged in CB-2% and IPA-2% samples can be attributed to the presence of different conductive phases.^42^

**Fig. 1:**
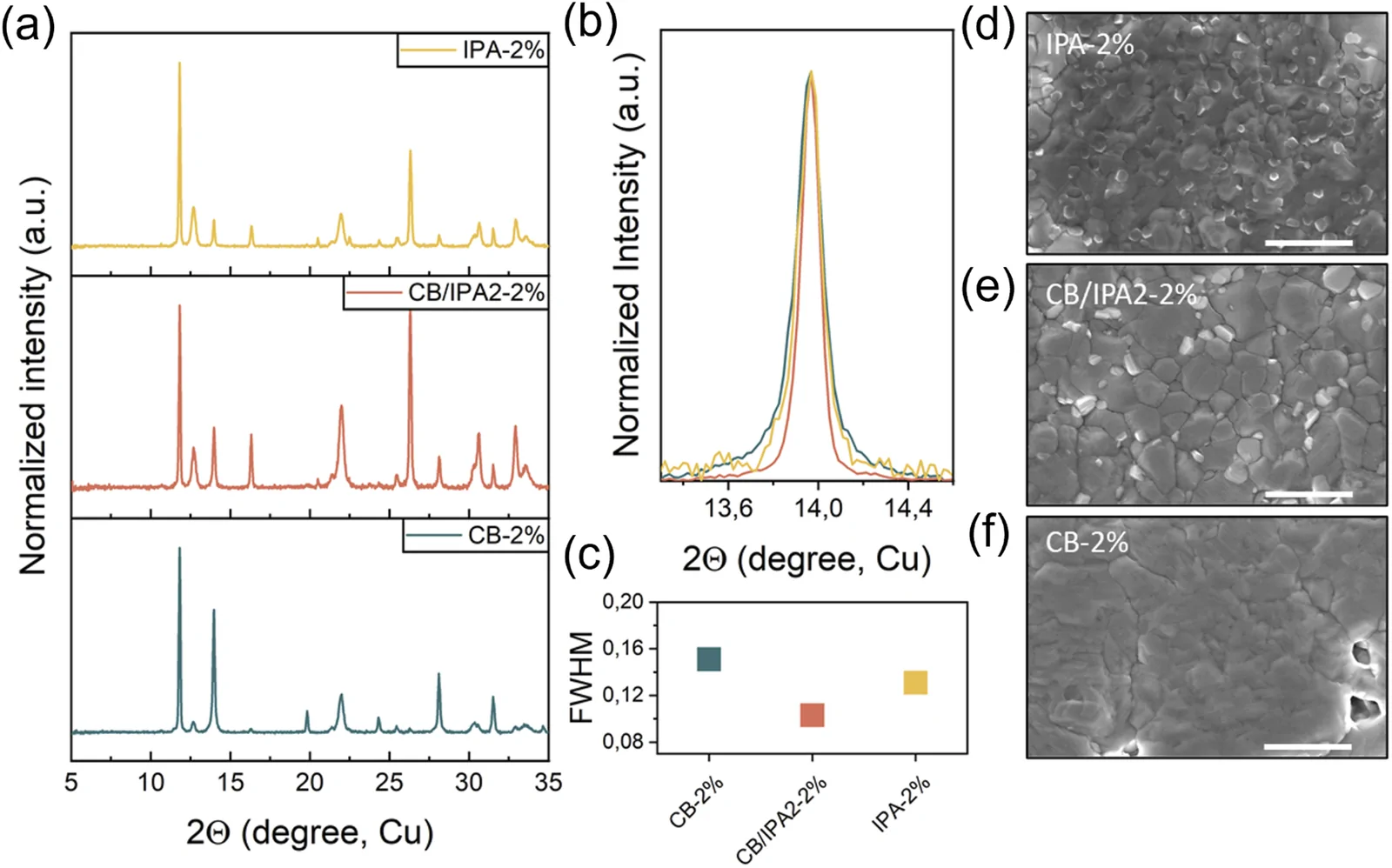
(a) X-ray diffraction pattern of FAPbI_3_ thin films with 2% Cs processed with CB (green line), CB/IPA2 (red line) and IPA (yellow line). (b) Enlarged view of the diffraction peak related to the (100) Miller plane of CB-2% (green line), CB/IPA2-2% (red line) and IPA-2% (yellow line) samples. (c) Full width at half maximum of the diffraction peak related to the (100) lattice plane of CB-2%, CB/IPA2-2% and IPA-2% samples. (d–f) SEM images of IPA-2%, CB/IPA2-2% and CB-2% thin films. The length of the scale bar is 1 µm.

To investigate the role of polarity in stabilizing the FAPbI_3_ α-phase, we tested our approach by replacing IPA with ethanol (EtOH), which has a higher polarity, and butanol (Bu*t*OH), which has a lower polarity. In the case of ethanol, a significant amount of the δ-phase remained in the films, whereas butanol resulted in a higher degree of conversion, comparable to that obtained with IPA (Fig. S5). Given the chemical similarity among ethanol, IPA, and butanol, and considering their different polarities, these results suggest that achieving an appropriate balance in the polarity of the antisolvent may play a central role in achieving a homogeneous phase conversion. Nevertheless, other solvent properties, such as hydrogen-bonding ability, miscibility, and evaporation kinetics, may also contribute to the observed stabilization.

To assess the influence of different antisolvent engineering strategies on the optoelectronic properties and microscale uniformity of FAPbI_3_ films, we performed hyperspectral photoluminescence (PL) mapping on CB-2%, CB/IPA2-2% and IPA-2% thin films. Fig. 2(a)–(c) present the broadband PL images of these films, with their corresponding PL intensity distribution plots shown in Fig. S6. Among all the films, the CB/IPA2-2% film exhibits the highest PL intensity and the most uniform emission, indicating superior film quality. This suggests that the CB/IPA2-2% formulation effectively minimizes non-radiative recombination losses. Fig. S7–S9 present a spectral analysis of the hyperspectral PL maps (center of mass maps), alongside white light reflection data. These analyses reveal two distinct types of microscale regions in both the CB-2% and IPA-2% films, but not in the CB/IPA2-2% film. Each region is characterized by variations in reflection intensity and PL spectral features. These differences are primarily attributed to local bandgap fluctuations, likely caused by heterogeneous Cs^+^ distribution within the films. Specifically, compared to the center emission peak at 810 nm in all films, Cs-deficient regions ptin the CB-2% film exhibit lower-energy emission peaks around 815 nm, while Cs-rich regions in the IPA-2% film show higher-energy emission peaks at approximately 804 nm. The Cs^+^ distribution within the perovskite layer was investigated by ToF-SIMS analysis of samples processed with either individual solvents or the binary solvent system.Fig. 2(d)–(f) shows that, when CB alone is used, Cs^+^ is significantly more concentrated near the surface of the perovskite film compared to the samples treated either with the binary solvent mixture or with pure IPA. Among these latter conditions, the binary solvent system provides the most conformal Cs^+^ distribution throughout the entire thickness of the perovskite layer. In contrast, the sample treated only with IPA still exhibits a less uniform profile, with a partial accumulation of Cs^+^ in the middle to bottom region, as highlighted in Fig. 2(g)–(i). These results indicate that the binary solvent approach facilitates uniform incorporation of Cs^+^ throughout the perovskite film, which may contribute to improved film homogeneity. It is worth clarifying that the conversion from the δ- to the α-phase was considered essentially complete based on the combined evidence from XRD and PL imaging. In the CB-2% and IPA-2% samples, the residual δ-phase is evidenced by the presence of its characteristic XRD peak and by non-emitting regions in the PL maps, respectively, whereas neither feature is observed for the CB/IPA2-2% sample. However, these measurements do not allow the residual δ-phase fraction to be quantitatively determined; therefore, “complete conversion” here refers to the absence of a detectable δ-phase within the sensitivity of the employed characterization techniques.

**Fig. 2:**
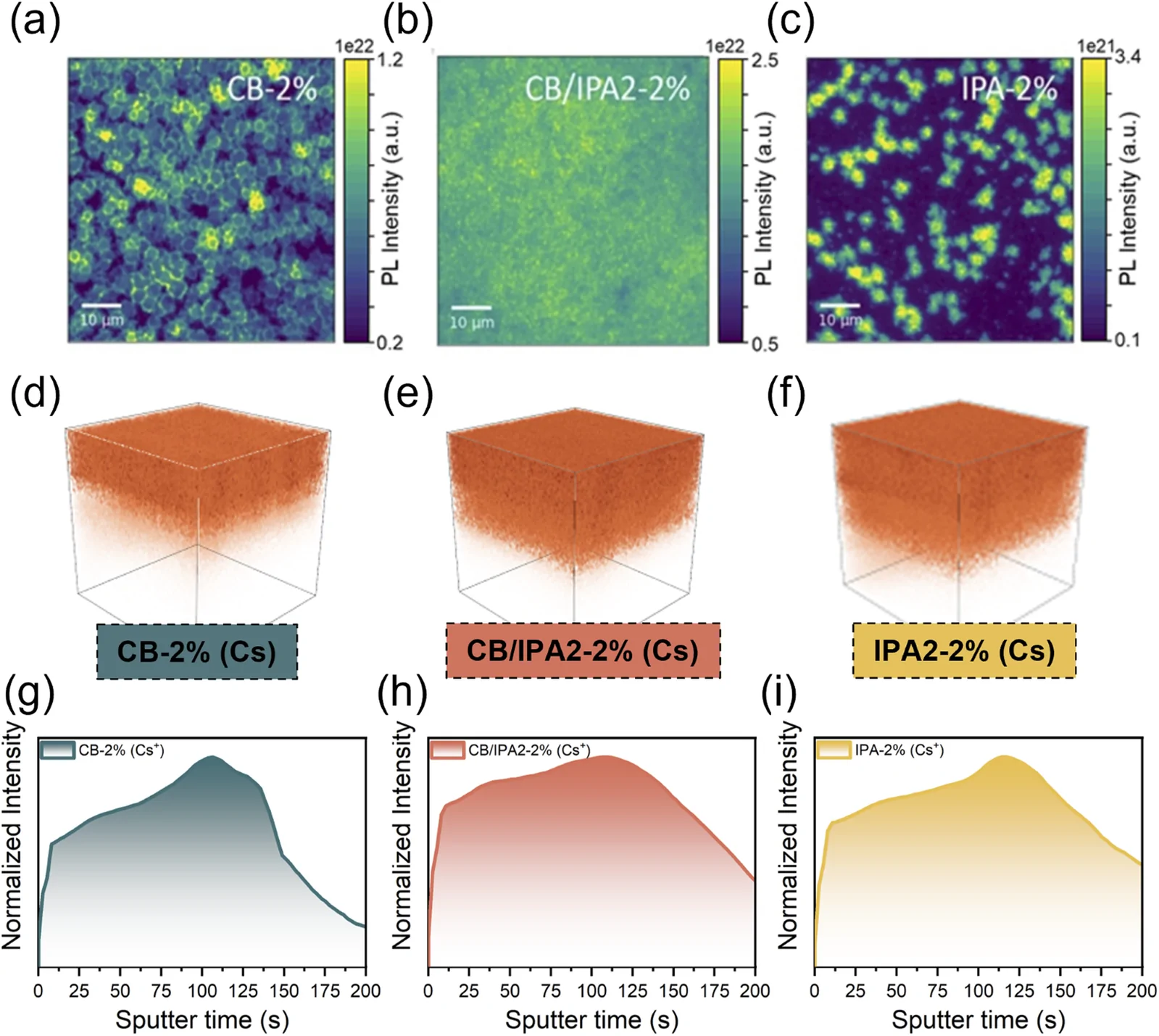
(a–c) Broadband PL maps of CB-2%, CB/IPA2-2% and IPA-2% perovskite films on ITO/SAM substrates. 3D Cs distributions, extrapolated from ToF-SIMS analyses, of (d) CB-2%, (e) CB/IPA2-2% and (f) IPA-2%. (g)–(i) Time-of-flight secondary ion mass spectrometry (ToF-SIMS) profiles of Cs^+^ within CB-2%, CB/IPA2-2% and IPA-2% samples.

The role of IPA in promoting phase homogeneity was further investigated by comparing the solubility of the organic precursors, CsI and FAI, in pristine chlorobenzene and in the CB/IPA binary solvent mixture. As expected, both salts exhibited negligible solubility in pristine CB, owing to its low polarity. In contrast, the addition of IPA markedly increased the solubility of FAI, while only minimally affecting that of CsI (Fig. S10). Taken together with the PL mapping and ToF-SIMS results discussed above, these findings support our hypothesis that the presence of IPA, in which FA^+^ is partially soluble, facilitates the redistribution of FAI during the antisolvent step. This enhanced FAI redistribution may, in turn, favour a more homogeneous distribution of Cs^+^ throughout the film, preventing its local accumulation and associated compositional inhomogeneities. Since Cs^+^ plays a key role in stabilizing the α-phase at low processing temperatures, its more homogeneous distribution is expected to facilitate uniform phase conversion throughout the film, ultimately enabling the conversion toward the α-phase (Scheme 1). To assess whether the antisolvent composition affects the precursor stoichiometry, energy-dispersive X-ray spectroscopy (EDX) measurements were performed to determine the Cs/Pb ratio in the resulting films. As shown in Fig. S11, the measured Cs/Pb ratio is approximately 3%, slightly higher than the nominal Cs concentration of 2%. This small deviation may be related to the different solubilities of FAI and CsI in the antisolvent mixture. In particular, the higher solubility of FAI may lead to a preferential removal of FA-containing species during the antisolvent treatment, resulting in a slight relative enrichment of Cs^+^ in the resulting film.

**Scheme 1:**
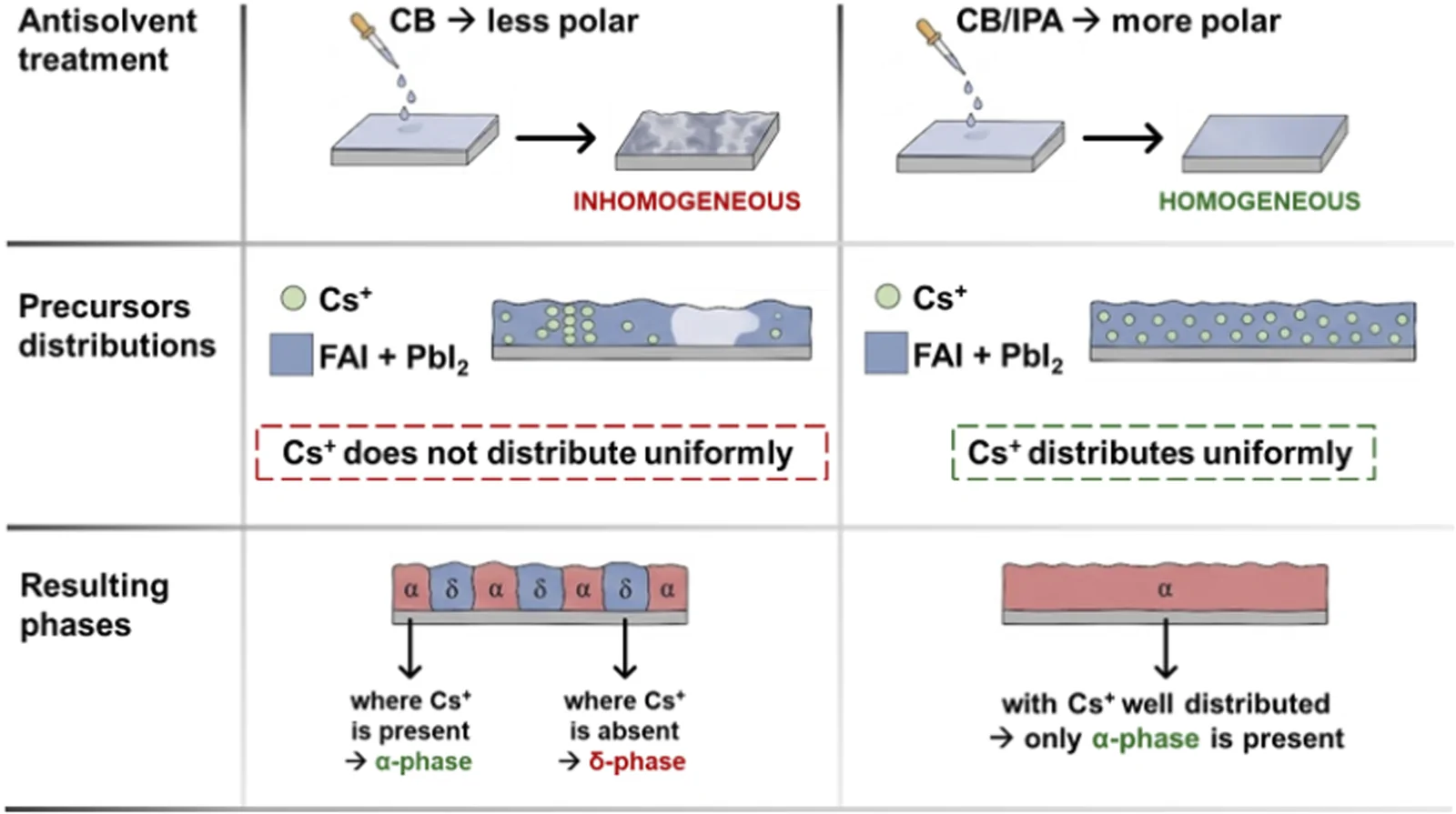
Schematic illustration of the proposed mechanism for stabilizing the α-FAPbI_3_ phase.

In parallel, we measured the carrier lifetime by time-resolved photoluminescence (TRPL) to probe bulk recombination in CB-2%, IPA-2%, and CB/IPA2-2% perovskite thin films. As shown in Fig. S12, the CB/IPA2-2% sample exhibits longer *τ*_1_ and *τ*_2_ than the single-solvent counterparts, indicating reduced non-radiative losses and improved bulk quality.^43^ This improvement is likely attributable to the higher film homogeneity of the binary-solvent sample with respect to samples treated with single solvents, consistent with the PL imaging. Given the quality of CB/IPA2-2% samples, we integrated the thin films into solar cell devices in a p–i–n configuration with the stack reported in Fig. 3(a). Despite the non-optimal optical characteristics of CB-2% and IPA-2% thin films due to their poor morphology and phase heterogeneity, we decided to fabricate devices as comparative examples. InFig. 3(b)–(e), box charts showing the distributions of short-circuit current (*J*_sc_), open-circuit voltage (*V*_oc_), Fill Factor (FF) and PCE are presented. As expected from the characterization reported above, the photovoltaic parameters of the CB/IPA2-2% sample were higher, and more reproducible, than those of CB-2% and IPA-2%. Indeed, the optimal FAPbI_3_ processing resulted in a *V*_oc_ of 1.08 V, a *J*_sc_ of 24.2 mA cm^−2^, an FF of 83.8% and a PCE of 21.8%. Additionally, the comparison between forward and reverse scans, reported in Fig. S13, shows a decrease in the average Hysteresis Index (HI) for the CB/IPA2-2% sample. Note that no passivation layer or antireflection coating was employed in the device fabrication. Fig. 3(f) displays the characteristic current–voltage curves of the three different samples. As mentioned above, the better photovoltaic properties of the CB/IPA2-2% sample with respect to the CB-2% and IPA-2% ones are attributable to both phase homogeneity and better morphology of the sample that has been processed with the binary mixture of solvents.^44,45^

**Fig. 3:**
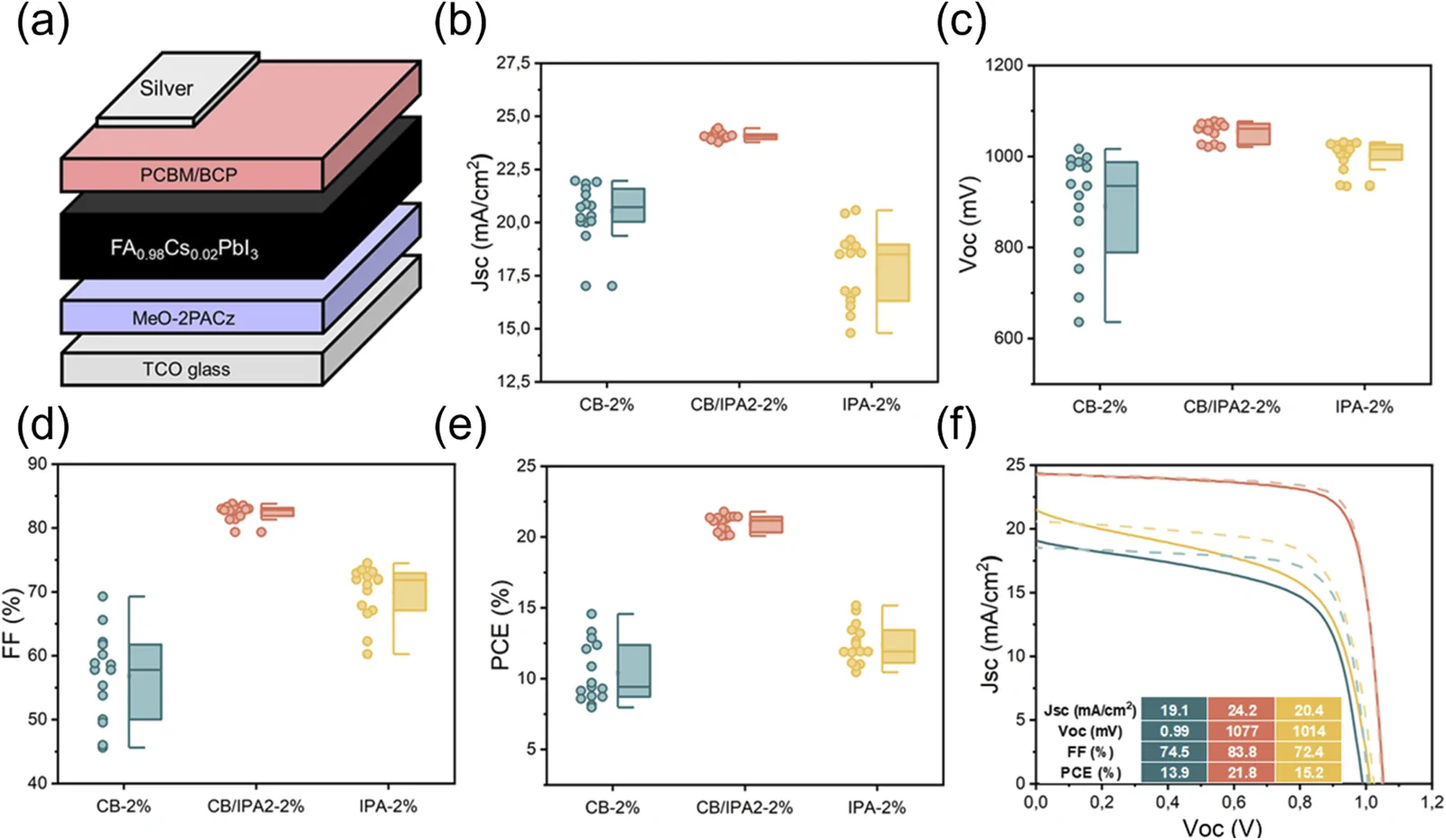
(a) Schematic representation of the fabricated p–i–n PSCs. Box charts of (b) *J*_sc_, (c) *V*_oc_, (d) FF and (e) PCE of FAPbI_3_ PSCs with 2% Cs fabricated with different antisolvent compositions. The horizontal lines in the box charts indicate the mean value. (f) Current density–voltage (*J*–*V*) curves in forward (For.) and reverse (Rev.) scans of CB-2% (green lines), CB/IPA2-2% (red lines) and IPA-2% thin films (yellow lines). For each condition, the PV parameters of 15 devices fabricated within the same batch are reported.

We subsequently evaluated the performance of our MA-free FAPbI_3_ strategy in comparison with the conventional MACl-based approach by fabricating reference devices incorporating 25 mol% MACl, as commonly reported in the literature.^46,47^ These devices are hereafter referred to as MACl-FAPbI_3_ and were used as a reference for comparison with the MA-free FAPbI_3_ devices. Fig. S14 and S15 show the comparison between PV parameter distributions and MPP stability (4 samples per condition) of MA-free and MACl-FAPbI_3_, respectively. Remarkably, the stability, tested under a nitrogen atmosphere, as well as the PV parameters and reproducibility were improved for CB/IPA2-2% devices. Notably, unlike the MA-free FAPbI_3_ case reported in Fig. 3, the binary solvent system led to a decrease in all PV parameters in the case of MACl-FAPbI_3_ devices. These results further support our conclusion that the binary solvent system is beneficial only when a small amount of cesium is added to the perovskite composition.

Lastly, given that there is no consensus in the community regarding the complete or incomplete volatilization of the MACl additive during FAPbI_3_ annealing, we decided to compare the optical and structural properties of the CB/IPA2-2% sample with MACl-FAPbI_3_ thin films. Note that, although the use of MACl enables the conversion of FAPbI_3_ from the δ- to the α-phase, the MACl-FAPbI_3_ sample underwent rapid degradation upon exposure to ambient conditions outside the glovebox if annealed at 100 °C; for that reason, we increased the annealing temperature to 150 °C. Fig. 4(a) and (b) show the Tauc plot analysis used to determine the band gap of the perovskite films.^48,49^ As observed, the band gap decreases when moving from the MACl-FAPbI_3_ to the MA-free FAPbI_3_ sample. Three samples were tested for each condition, and the distribution of the extracted band-gap values is reported as box plots in Fig. 4(c). The same trend was confirmed by comparing the steady-state photoluminescence spectra of the two samples (Fig. 4(d)–(f)). Indeed, the *λ*_max_ position was systematically red-shifted for the CB/IPA2-2% sample compared with the MACl-FAPbI_3_ one. This result was further corroborated by incident photon-to-current conversion efficiency (IPCE) measurements reported in Fig. S16. The MA-free sample exhibited a shift of the IPCE cut-off edge toward higher wavelengths, consistent with its lower band gap.^50^

**Fig. 4:**
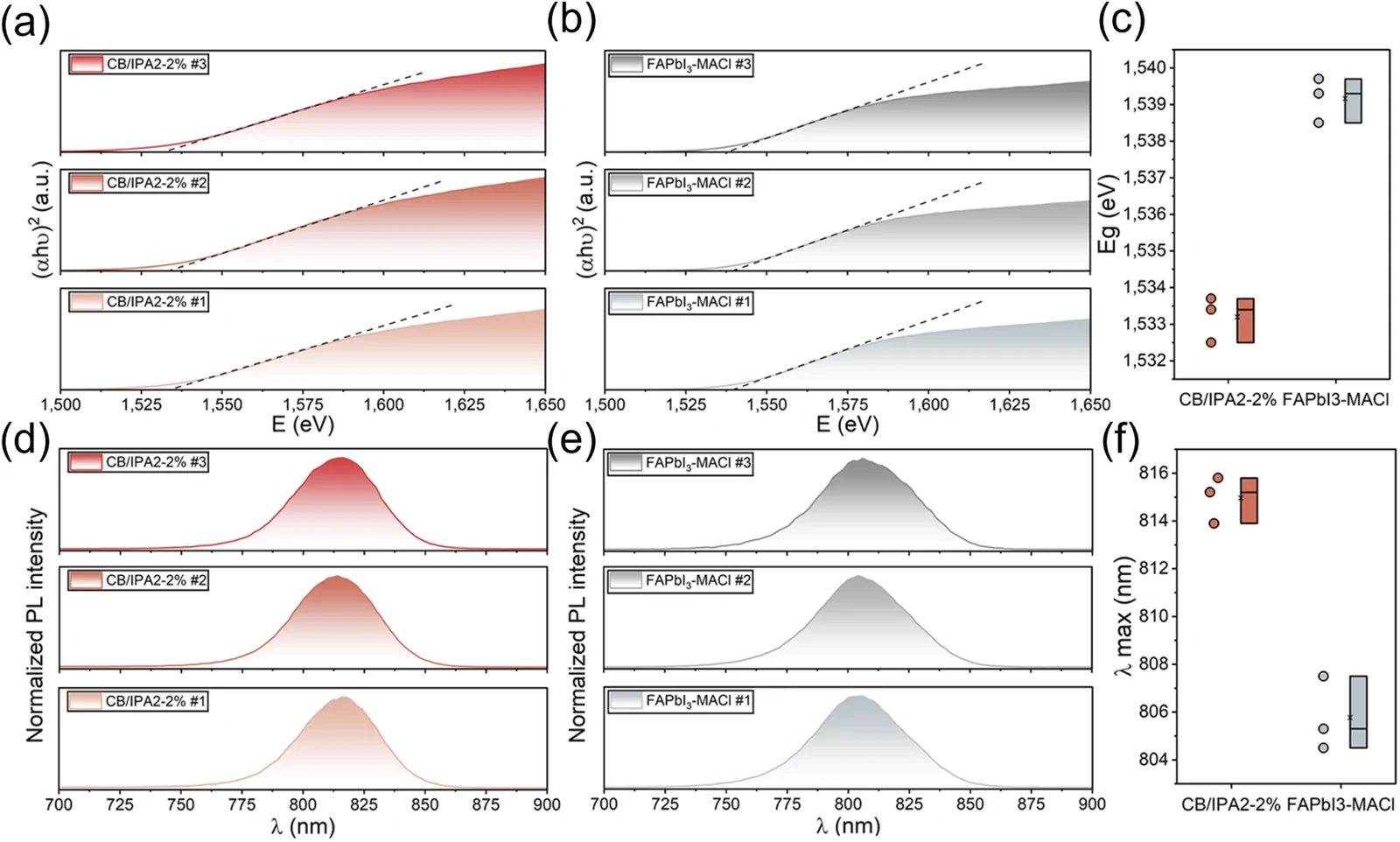
Tauc plots of three (a) MACl-FAPbI_3_ and (b) three CB/IPA2-2% thin films. (c) Box charts showing the distribution of the optical band gap values extrapolated from the corresponding Tauc plots. (d) and (e) Photoluminescence (PL) spectra of three MACl-FAPbI_3_ and three CB/IPA2-2% thin films, respectively. (f) Box chart showing the distribution of the PL peak wavelength maximum extracted from the corresponding PL spectra.

To better understand the origin of this optical difference, we performed accurate XRD measurements and refined the lattice parameters (Fig. S17). From the analysis of the XRD patterns, two significant differences emerged. The first one (schematized in Fig. S18) is related to the different crystallographic orientation of the two samples, completely along the [100] crystallographic direction for MACl-FAPbI_3_ (in accordance with the literature),^51,52^ while CB/IPA2-2% presented an additional predominant orientation along the [*h*_*n*_*k*_*n*_0] crystallographic direction. The second difference involved lattice parameters, as extrapolated by refining the XRD pattern.^53^ Indeed, MACl-FAPbI_3_ showed a contraction of lattice constants with respect to those of CB/IPA2-2%. This result, together with the change in the band gap reported in Fig. 4, suggests an incorporation of a small amount of methylammonium with a consequent increase in the band gap of the material and a contraction of the lattice constants (due to the smaller size of methylammonium with respect to formamidinium) in MACl-FAPbI_3_.^54^

## Conclusions

In summary, we have demonstrated a simple yet effective strategy to stabilize the black phase of FAPbI_3_ at 100 °C by introducing a minimal amount of Cs^+^ in the precursor solution, just sufficient to ensure complete conversion from the non-perovskite δ-phase, combined with a modified antisolvent engineering approach. In particular, the use of a binary antisolvent system, obtained by adding a controlled fraction of isopropanol to conventional chlorobenzene, was found to be crucial in achieving a homogeneous and controlled yellow-to-black phase transition of FAPbI_3_ at 100 °C. Beyond improving phase conversion, this solvent engineering also plays a key role in regulating the spatial distribution of cesium within the perovskite layer. As evidenced by ToF-SIMS analysis, chlorobenzene-only treatment leads to a pronounced accumulation of Cs^+^ near the surface of the film, indicating a highly non-uniform incorporation profile. In contrast, the addition of isopropanol results in a significantly more uniform cesium incorporation throughout the film thickness, consistent with a more homogeneous crystallization process and a controlled incorporation of the cation into the perovskite lattice as suggested from PL mapping analysis. Importantly, unlike common strategies that rely on the use of external additives or high concentrations of MA-based additives in the precursor solution, with our method we achieve an intrinsic stabilization of the perovskite structure. As a result, our films exhibit an optical band gap closer to that of pristine FAPbI_3_. Overall, this approach provides a promising pathway for the low-temperature fabrication of MA-free perovskite solar cells, combining α-phase stability, controlled ionic distribution, and preservation of the narrow band gap of FAPbI_3_.

## Author contributions

Conceptualization: RP, SF, and GG. Methodology: RP, SF, and AS. Investigation: RP, SF, WX, SM, SM, AS, RS, MD, MF, LH, and TX. Formal analysis: RP, SF, WX, SM, MF, SM, RS, and TX. Supervision: BMG, CQM, AO, CJB, SS, and GG. Writing – original draft: RP and WX. Writing – review & editing: RP, SF, WX, SM, SM, AS, RS, MD, LH, TX, BMG, CQM, AO, SS, CJB, and GG.

## Conflicts of interest

There are no conflicts to declare.

## Supplementary Material

EL-OLF-D6EL00108D-s001

## Data Availability

The data that support the findings of this study are available from the corresponding authors upon reasonable request. Supplementary information (SI) is available. See DOI: https://doi.org/10.1039/d6el00108d.
