# Supplementary material for: Cation redistribution enables low-temperature stabilization of MA-free FAPbI3
Source: EES Solar. 2026 Sep 30. Online ahead of print. doi: 10.1039/d6el00108d (PMC13660750; doi:10.1039/d6el00108d)
Supplement: Supplementary file 1 [file EL-OLF-D6EL00108D-s001.pdf]

Supplementary Information (SI) for EES Solar.
This journal is © The Royal Society of Chemistry

Supporting Information

Cation Redistribution Enables Low-Temperature Stabilization of MA-Free $FAPbI_3$

Riccardo Pallotta,[a,b] Simone Filippi,[a] Alice Scardina,[a] Weidong Xu,[c,d] Samuele Mattioni,[e,f] Mingjie Feng,[b] Sergio Marras,[g] Ruggero Sala,[a] Matteo Degani,[a] Luis E. Hueso,[e,h] Tianyu Xu,[i] Beatriz Martín-García,[e,h] Chang-Qi Ma,[i] Andres Osvet,[b] Samuel D. Stranks,[c,d] Christoph J. Brabec,[b,j] Giulia Grancini[a,]*

[a] Department of Chemistry and INSTM, University of Pavia, Via T. Taramelli 1,4, 27100 Pavia, Italy

[b] Institute of Materials for Electronics and Energy Technology (i-MEET), Department of Materials Science and Engineering, Friedrich-Alexander-Universität Erlangen-Nürnberg, Martensstraße 7, Erlangen 91058, Germany

[c] Department of chemical engineering & Biotechnology, University of Cambridge, Philippa Fawcett drive, Cambridge cB3 0AS, UK.

[d] Department of Physics, cavendish Laboratory, University of Cambridge, JJ Thomson Avenue, Cambridge cB3 0he, UK.

[e] CIC nanoGUNE BRTA, Tolosa Hiribidea, 76, 20018 Donostia-San Sebastián, Basque Country, Spain

[f] Departamento de Polímeros y Materiales Avanzados: Física, Química y Tecnología, University of the Basque Country (UPV/EHU), 20018 Donostia-San Sebastián, Basque Country, Spain

[g] Materials Characterization Facility, Istituto Italiano di Tecnologia Via Morego 30, 16163 Genova, Italy.

[h] IKERBASQUE-Basque Foundation for Science, 48009 Bilbao, Basque Country, Spain.

[i] *i*-Lab & Printable Electronics Research Center, Suzhou Institute of Nano-Tech and Nano-Bionics (SINANO), Chinese Academy of Sciences (CAS), Ruoshui Road 398, Suzhou 215123, China

[j] Helmholtz-Institute Erlangen-Nürnberg for Renewable Energy (HI ERN), Immerwahrstraße 2, Erlangen 91058 Germany

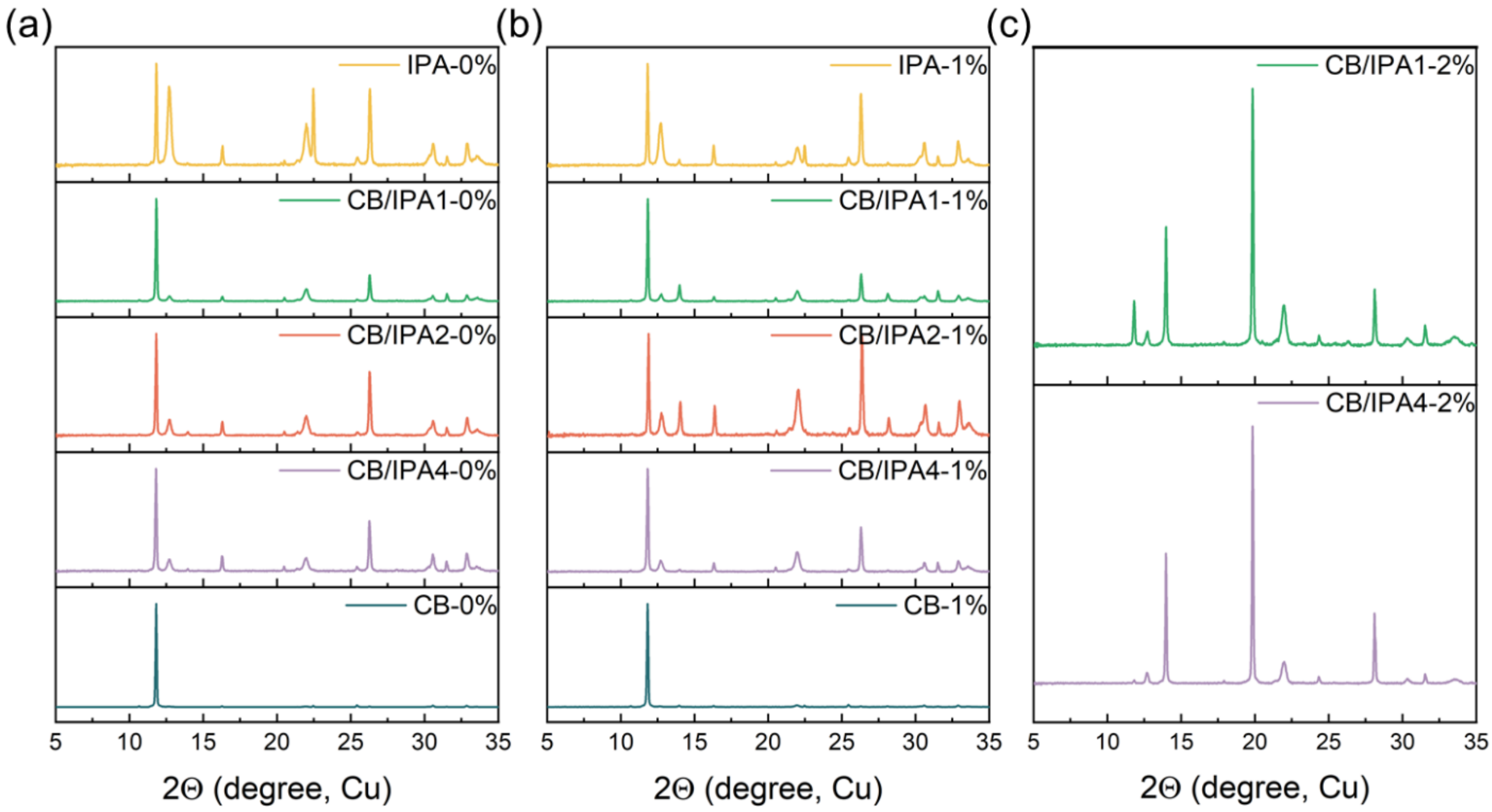

Figure S1. XRD patterns of perovskite thin films with a) 0% Cs b) 1% Cs c) 2% Cs and different antisolvent composition.

LEGEND:

- (red) δ-phase only
- (green) α-phase only
- (yellow) α/δ-phases

	0%Cs	1%Cs	2%Cs
CB	δ	δ	α/δ
CB/IPA 4	δ	δ	α/δ
CB/IPA2	δ	α/δ	α
CB/IPA1	δ	α/δ	α/δ
IPA	δ	α/δ	α/δ

Figure S2. Predominant $FAPbI_3$ crystal phase according to XRD patterns.

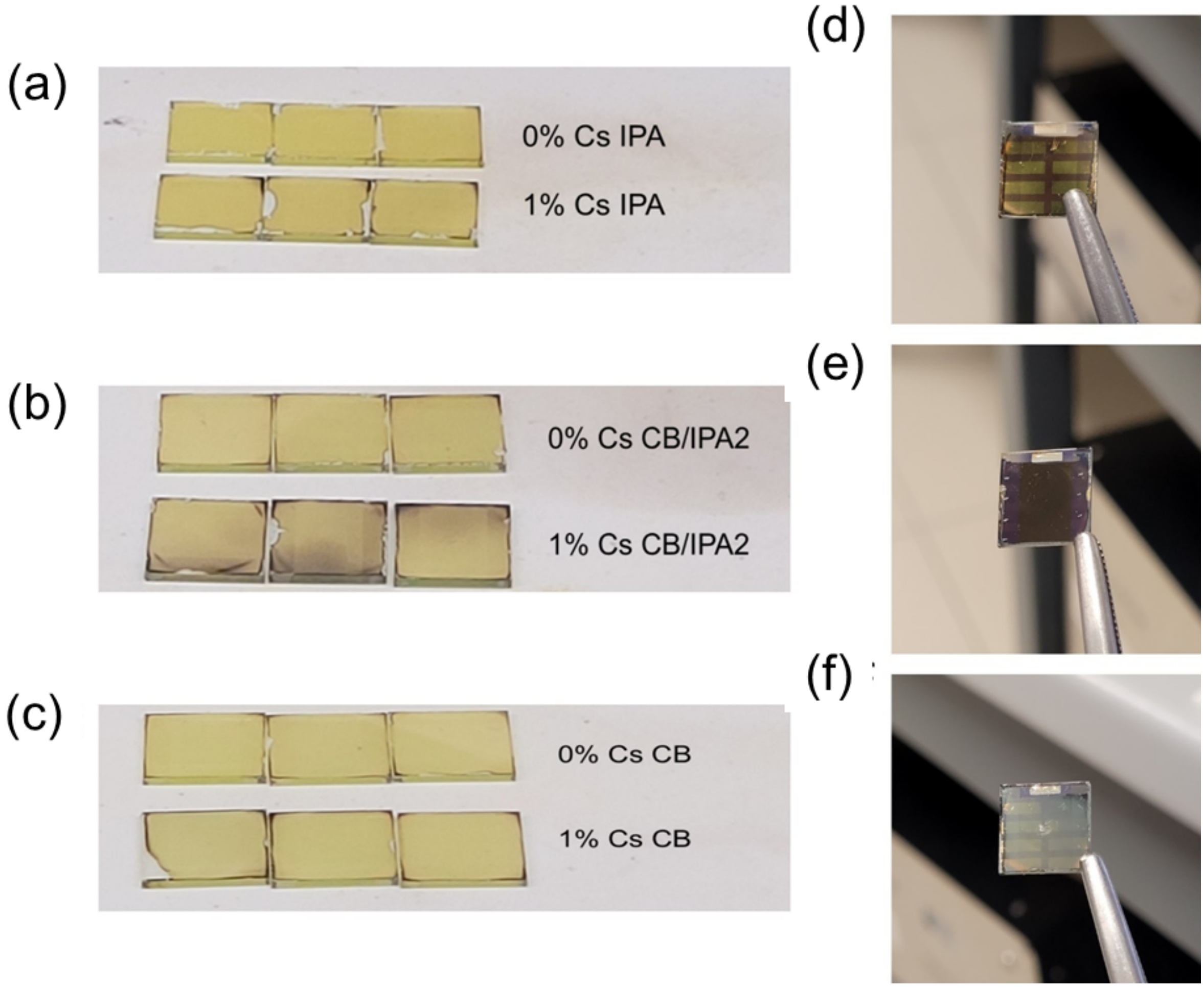

Figure S3. Visual appearance of $FAPbI_3$ thin films and devices processed at 100°C, on the left: 0% Cs and 1% Cs perovskite treated with a) IPA b) CB/IPA2 c) CB; on the right: 2% Cs $FAPbI_3$ treated with d) IPA e) CB/IPA2 and f) CB.

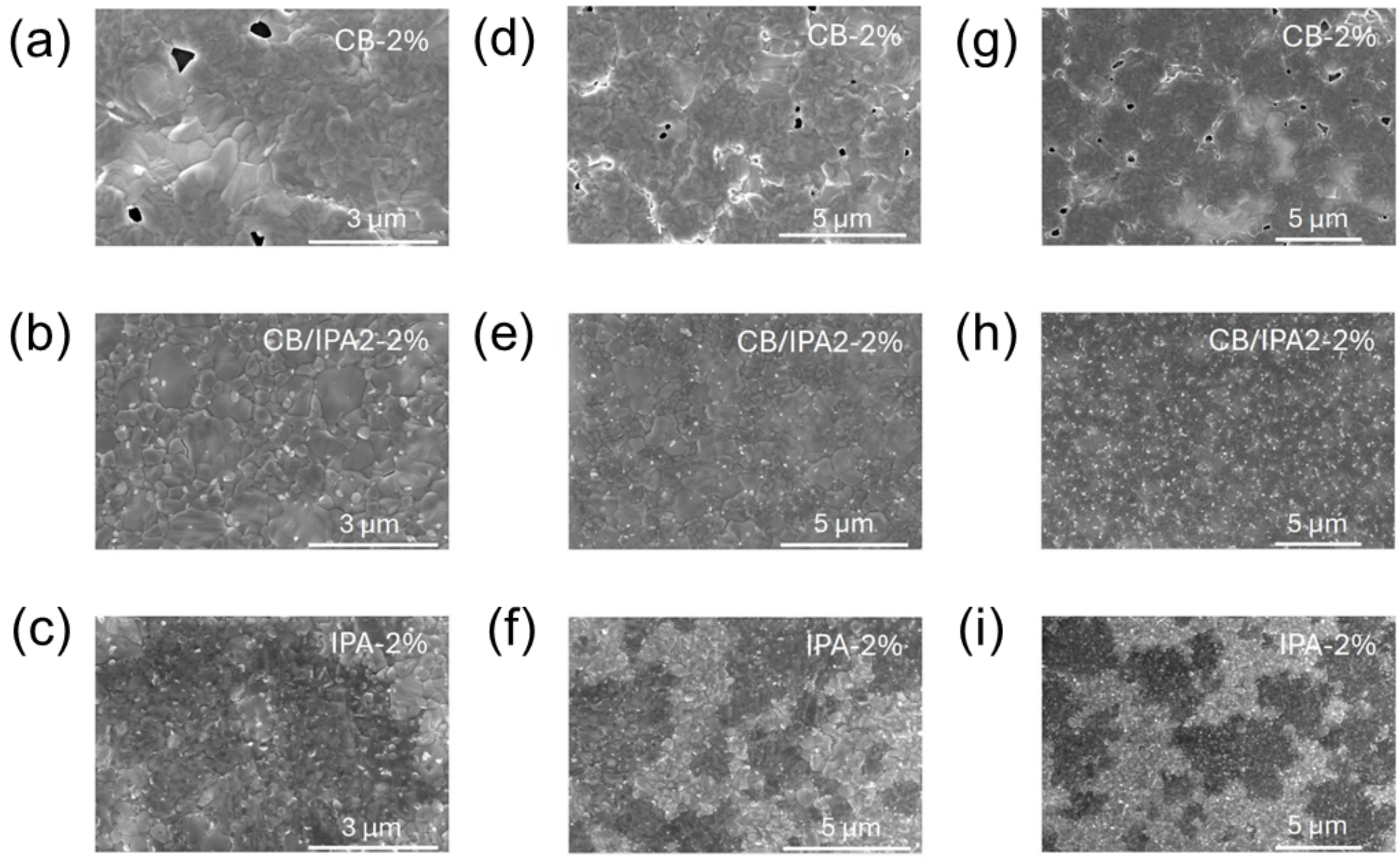

Figure S4. SEM images of (a-d-g) CB-2%, (b-e-h) CB/IPA2-2% and (c-f-i) IPA-2% samples at different magnitudes.

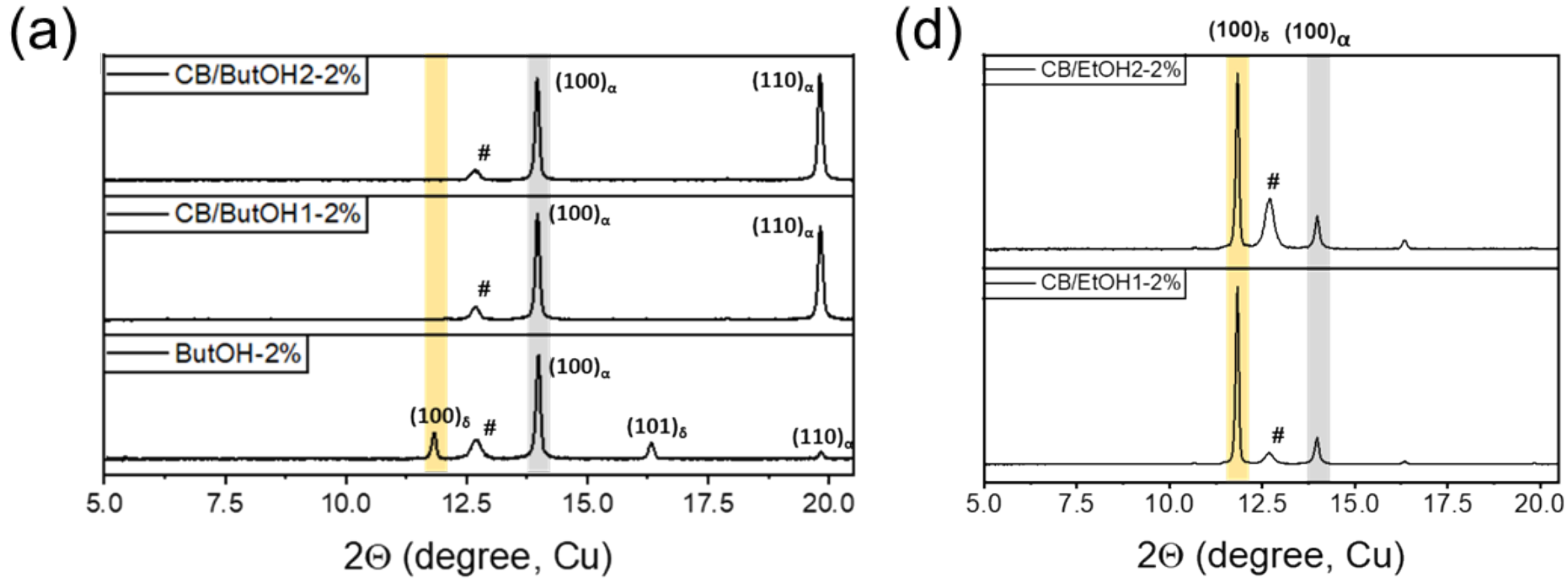

Figure S5. XRD pattern of MA-free samples processed with mixture between CB and butanol (ButOH) or ethanol (EtOH).

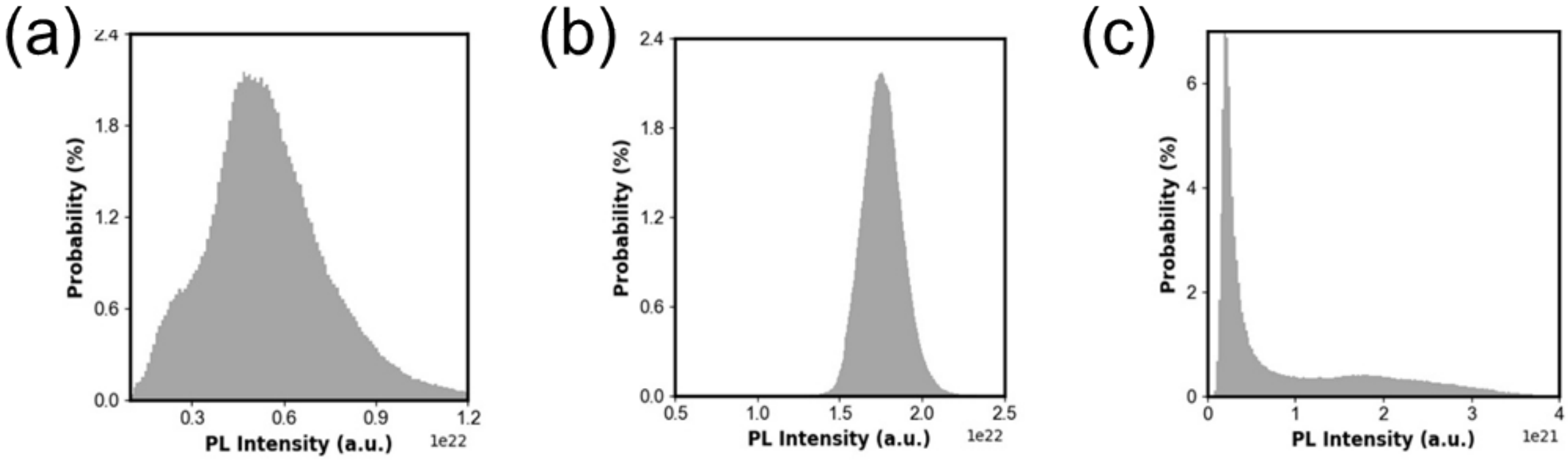

Figure S6. Statistical Distribution of broadband PL intensity of a) CB-2%, b) CB/IPA2-2% and c) IPA-2% thin films.

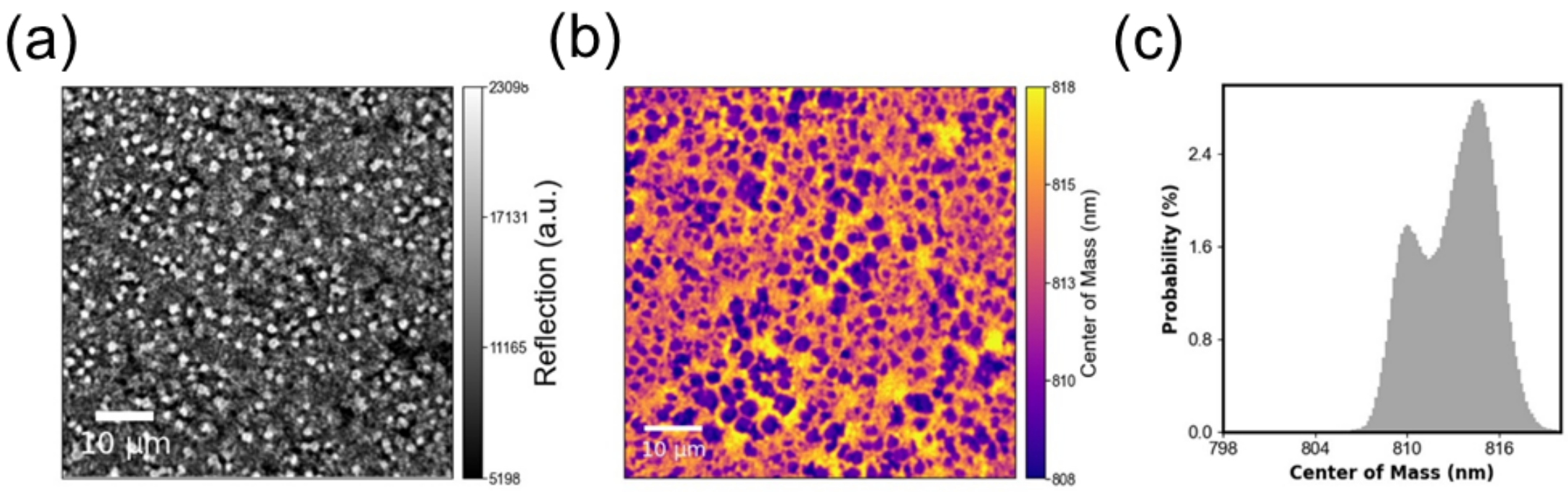

Figure S7. a) White light reflection b) spectral center of mass and c) statistical distribution of center of mass of CB-2% sample.

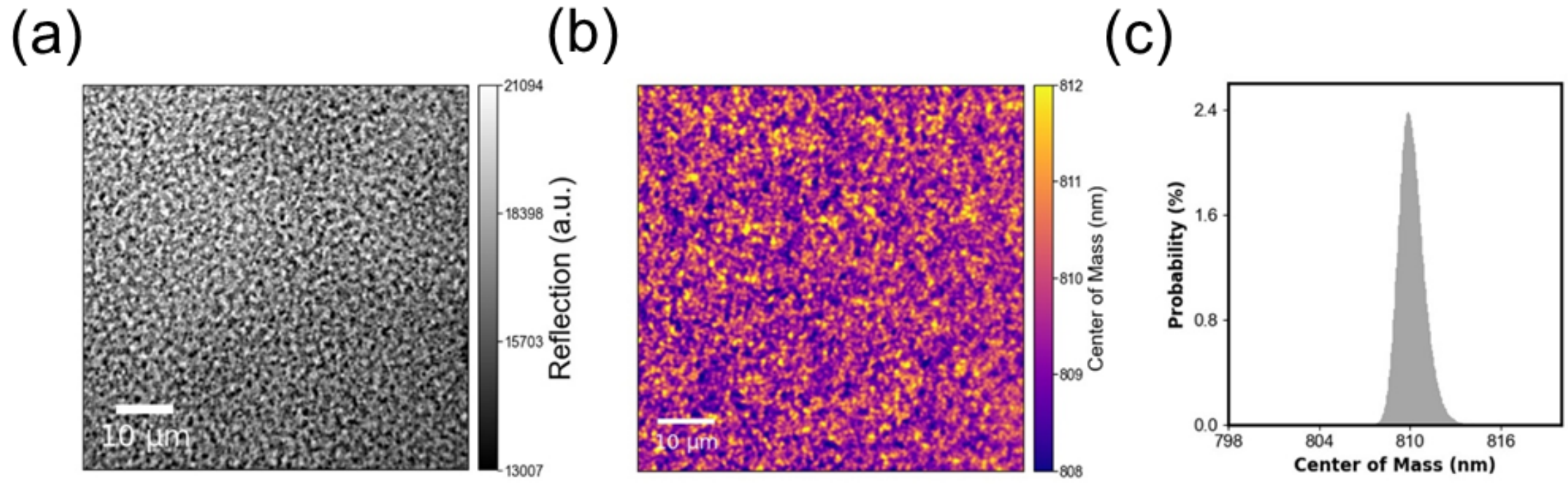

Figure S8. a) White light reflection b) spectral center of mass and c) statistical distribution of center of mass of CB/IPA2-2% sample

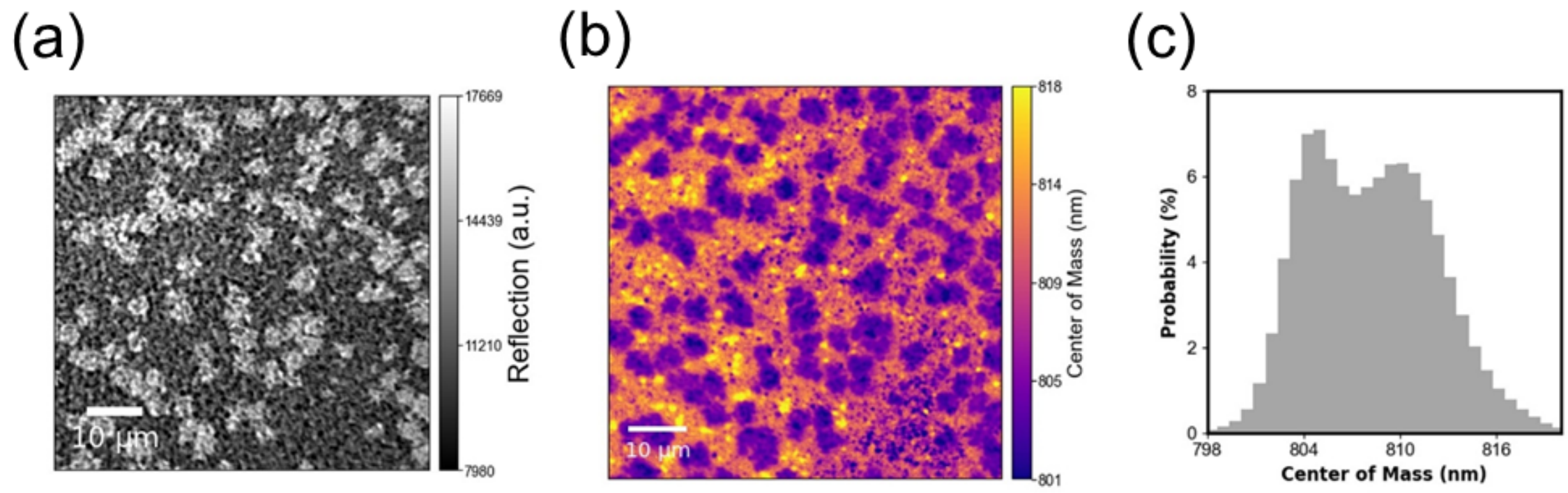

Figure S9. a) White light reflection b) spectral center of mass and c) statistical distribution of center of mass of IPA-2% sample

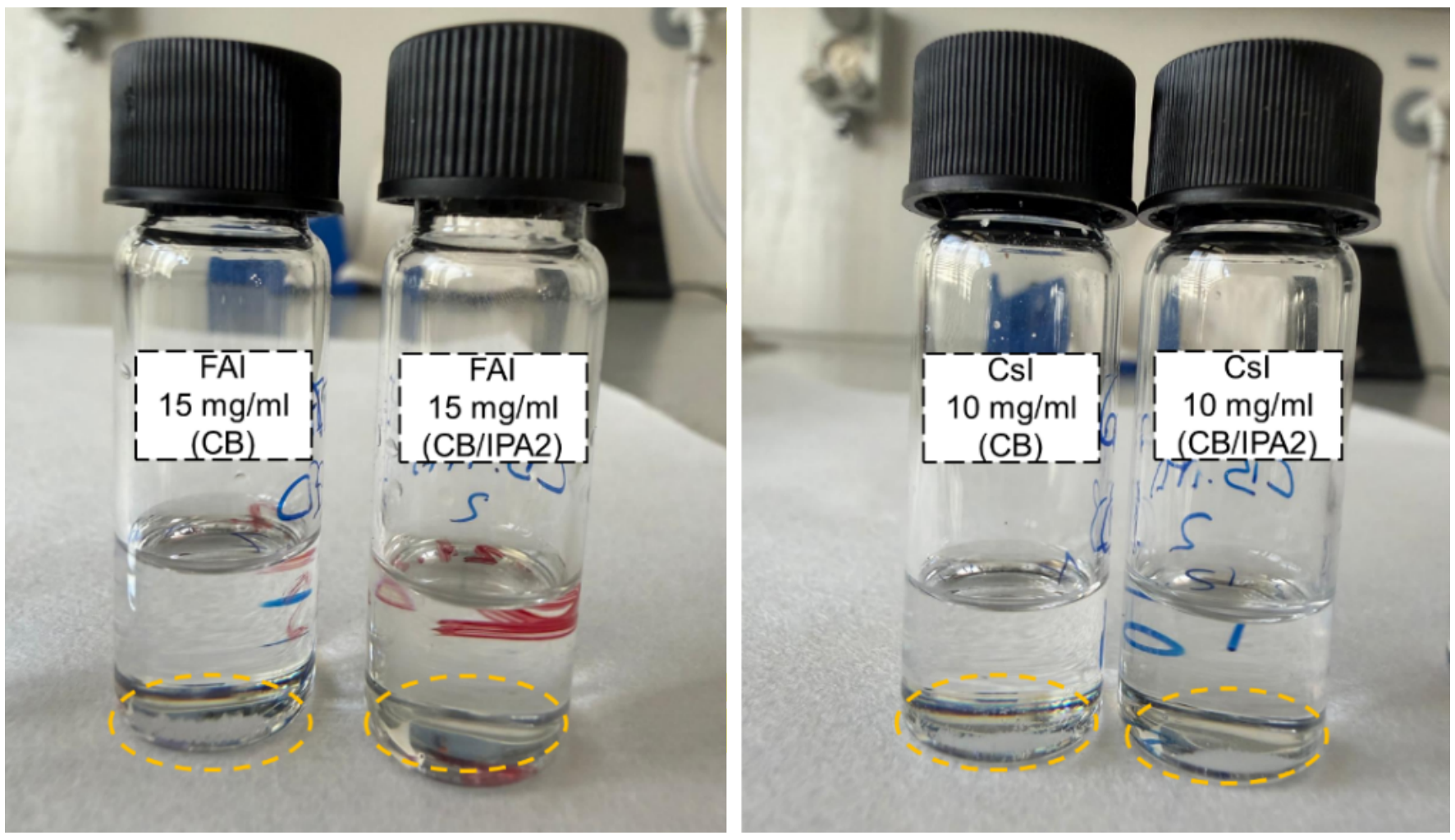

Figure S10. Solubility of FAI (15 mg mL^{-1}) and CsI (10 mg mL^{-1}) in pristine chlorobenzene (CB) and in the CB/IPA2 mixture.

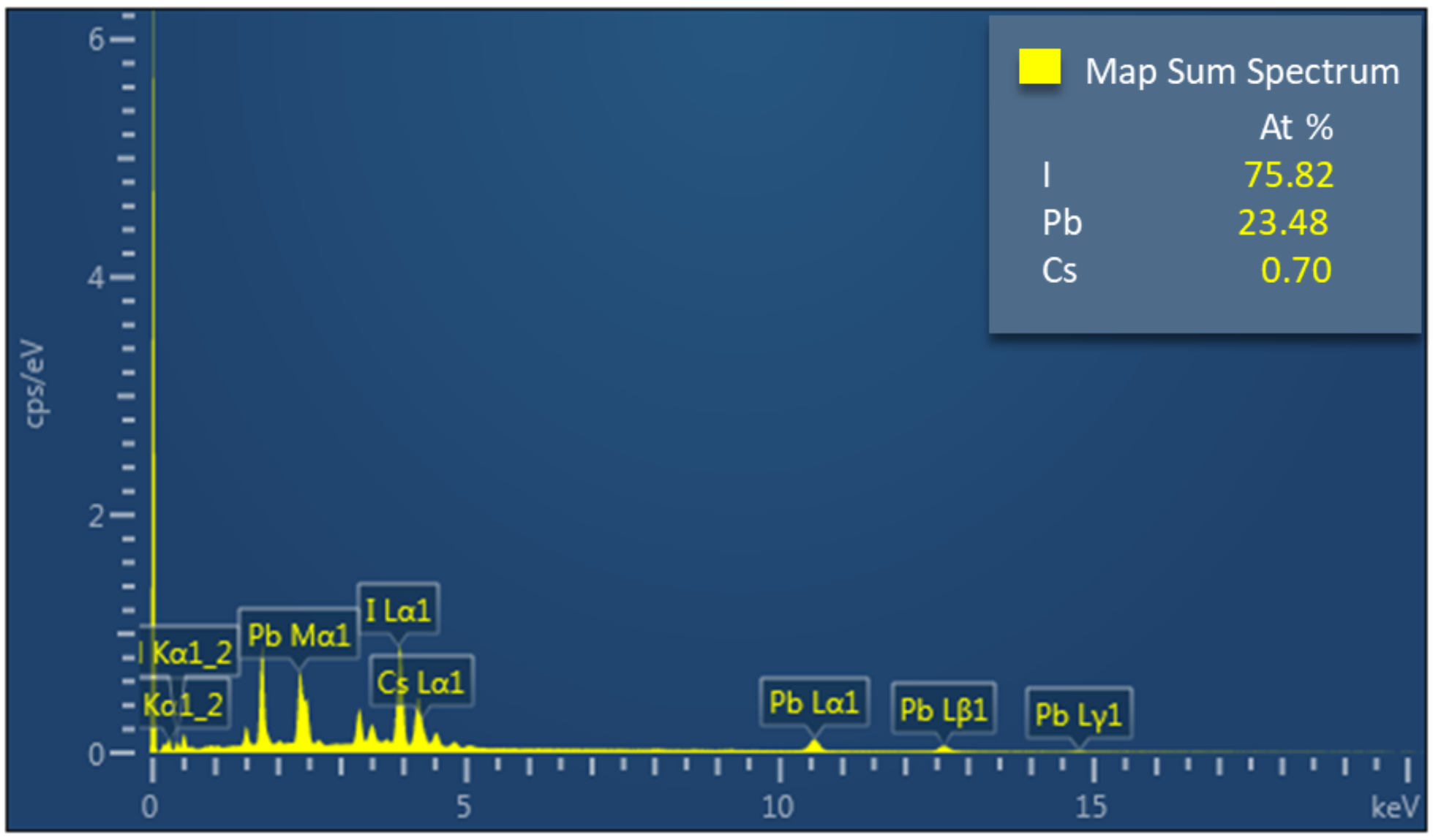

Figure S11. Energy-dispersive X-ray spectroscopy showing the atomic percentage of elements present in the CB/IPA2-2% sample.

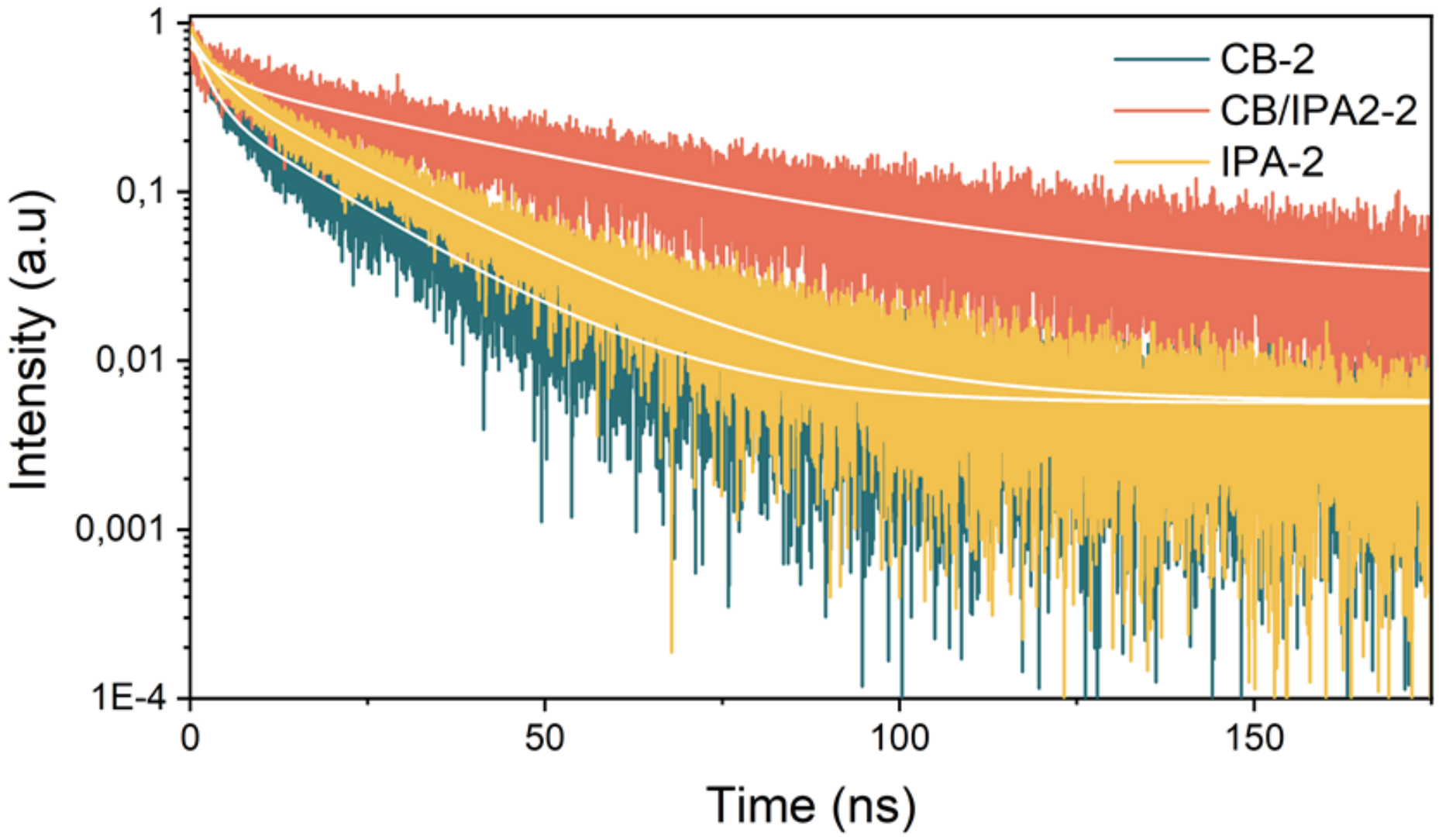

	A_1	τ_1	A_2	τ_2	C	τ_{eff}	R^2
**CB-2**	0.51	2.43	0.33	16.75	0.006	8.1	0.98
**CB/IPA-2**	0.26	3.99	0.44	43.36	0.027	28.7	0.92
**IPA-2**	0.38	3.36	0.46	19.95	0.008	12.4	0.99

Figure S12. Photoluminescence decay signal of CB-2% (green line), IPA-2% (yellow line) and CB/IPA2-2% (red line) samples deposited on glass. The results of the bi-exponential fitting curve are reported below.

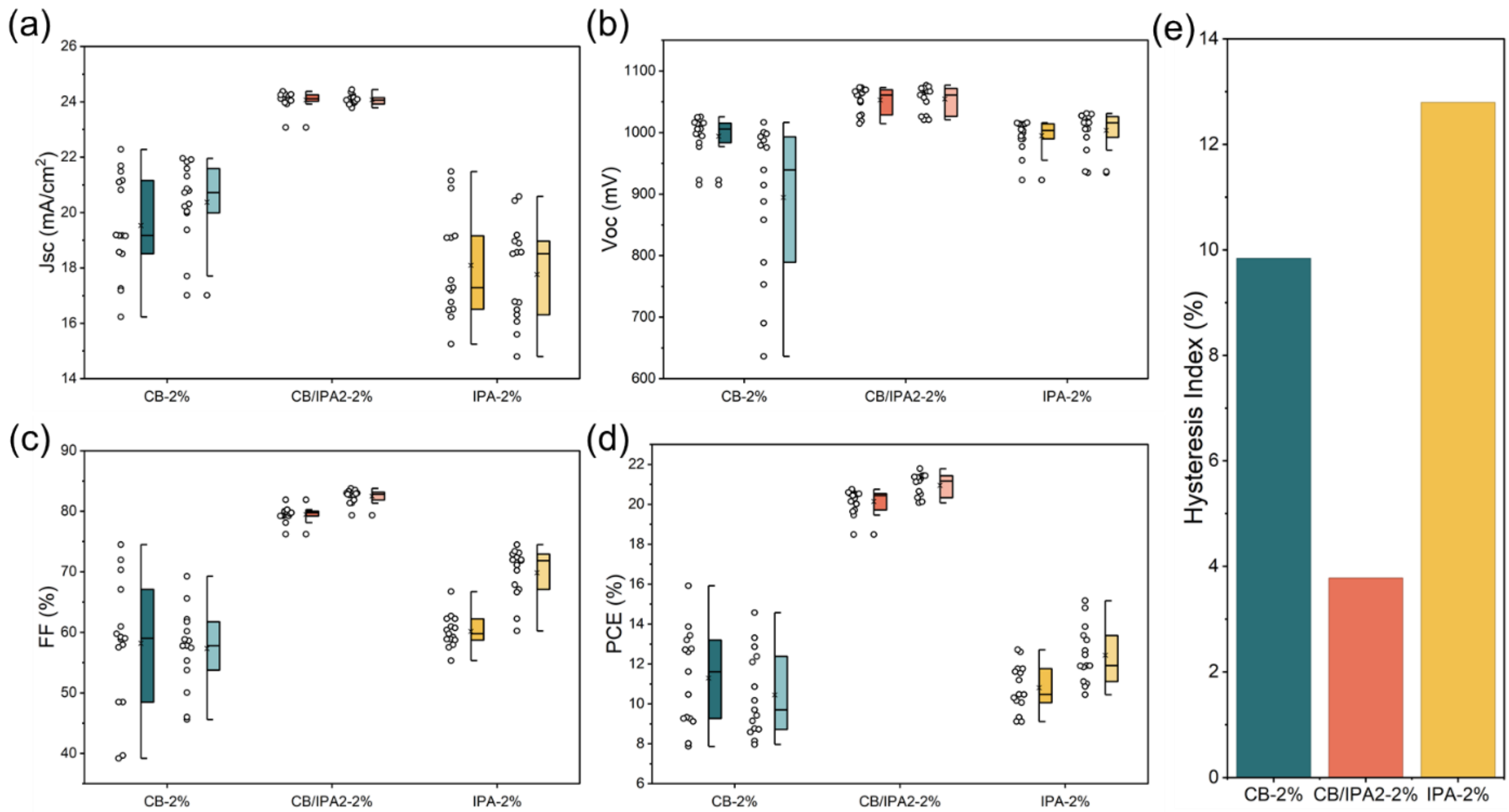

Figure S13. (a-d) Box charts showing the distributions of the photovoltaic parameters measured in the forward (dark colors) and reverse scans (light colors) for the CB-2%, CB/IPA2-2%, and IPA-2% samples. (e) Average hysteresis index calculated from the forward and reverse scans for the CB-2%, CB/IPA2-2%, and IPA-2% samples. For each condition, the PV parameters of 15 devices fabricated within the same batch are reported.

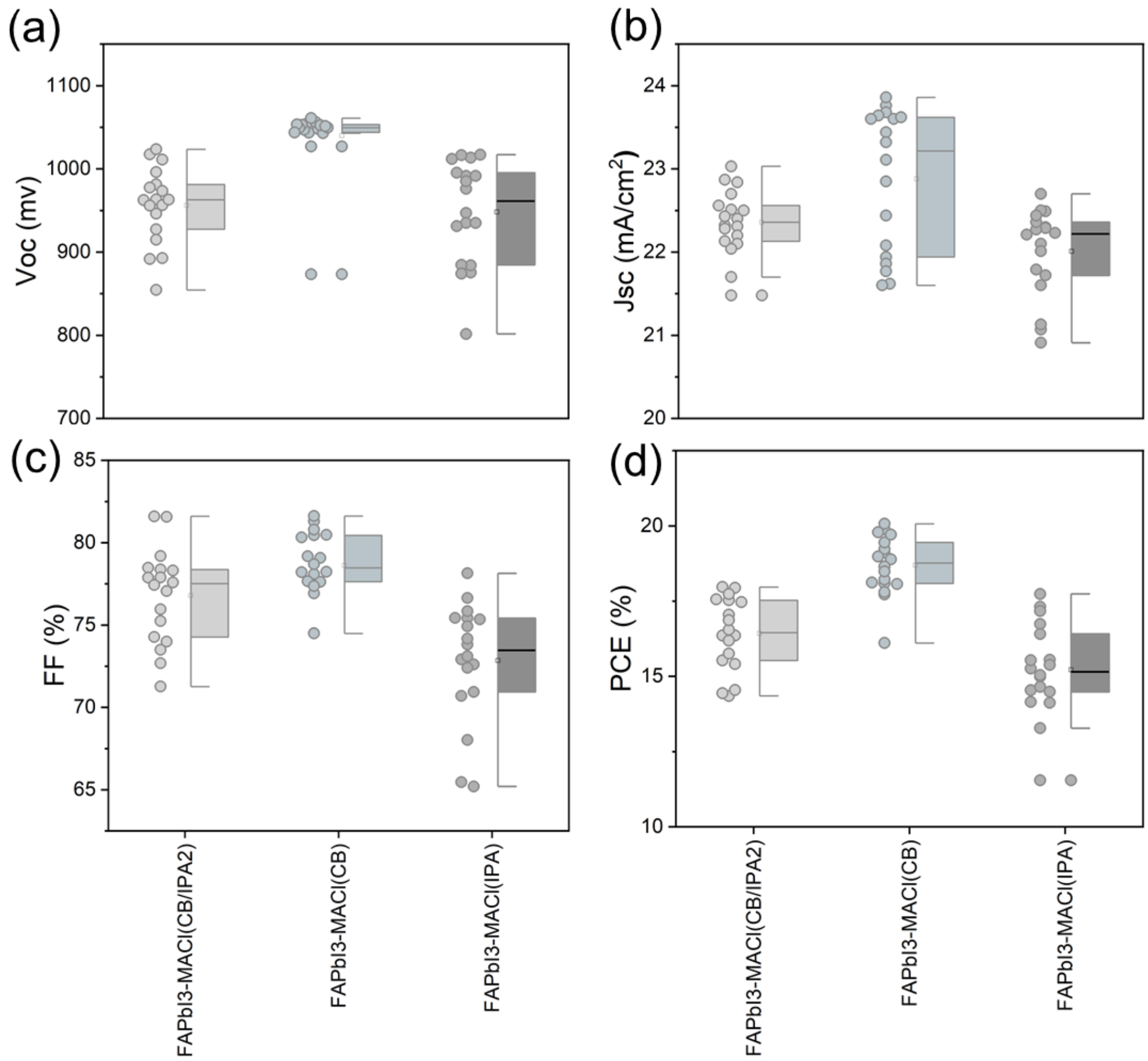

Figure S14. Box charts of J_{SC}, V_{OC}, FF and PCE of $FAPbI_3$-MACl treated with different solvents. The horizontal lines in box charts indicate the mean value. For each condition, the PV parameters of 18 devices fabricated within the same batch are reported.

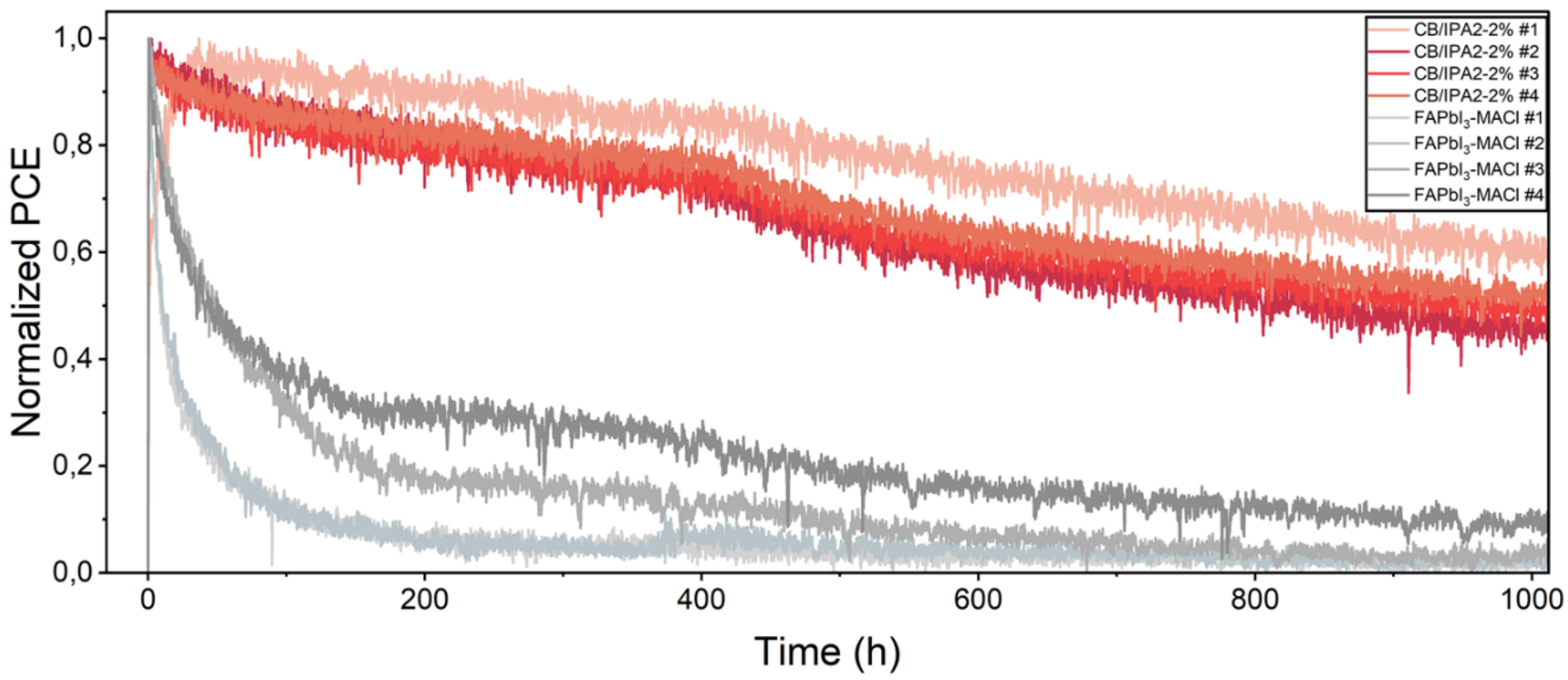

Figure S15. Maximum Power Point stability test for CB/IPA2-2% and $FAPbI_3$-MACl devices. 4 devices have been tested for each condition.

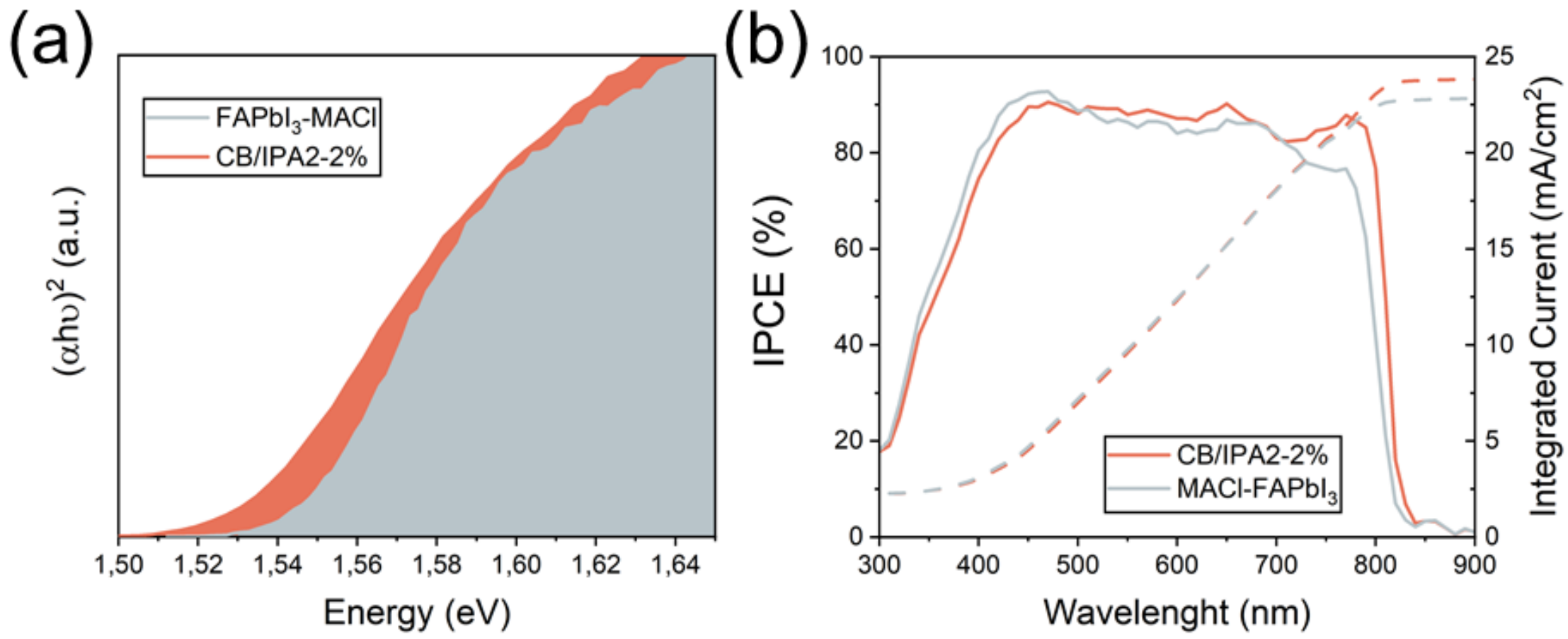

Figure 16. (a) Tauc Plot of MACl-$FAPbI_3$ (grey) and CB/IPA2-2% (red) thin films. (b) IPCE measurements of MACl-$FAPbI_3$ (grey line) and CB/IPA2-2% (red line) devices.

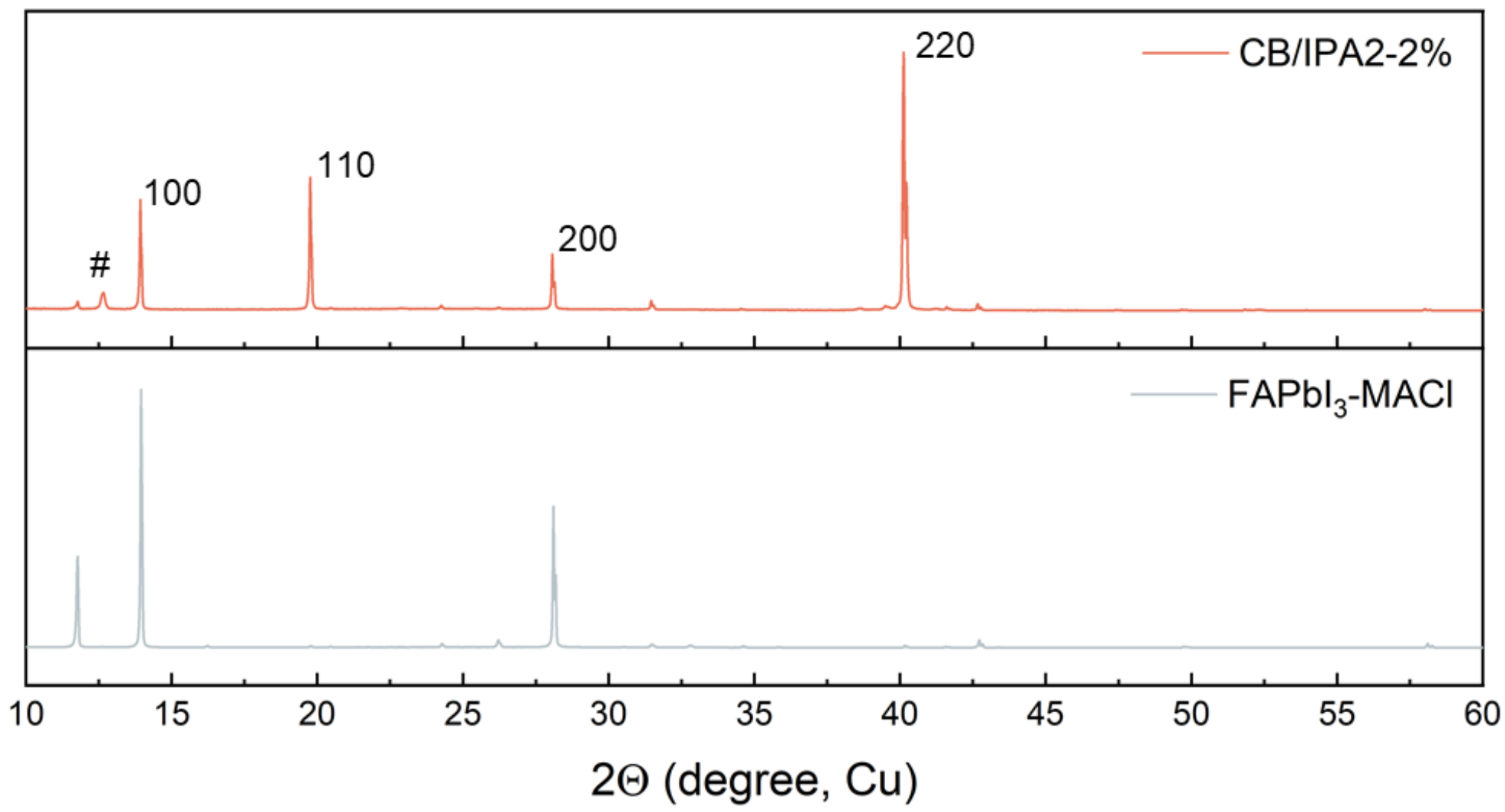

Space Group Pm-3m	lattice constants a, b, c (Å)	ESD (estimated standard deviation)
CB/IPA2-2%	6.3527	0.0004
FAPbI$_3$-MACl	6.3460	0.0005

Figure S17. Comparison of XRD patterns a) for CB/IPA2-2 and FAPbI$_3$-MACl samples, with indication of Miller indexes corresponding to the main peaks and b) estimated lattice constants with the respective estimated standard deviation.

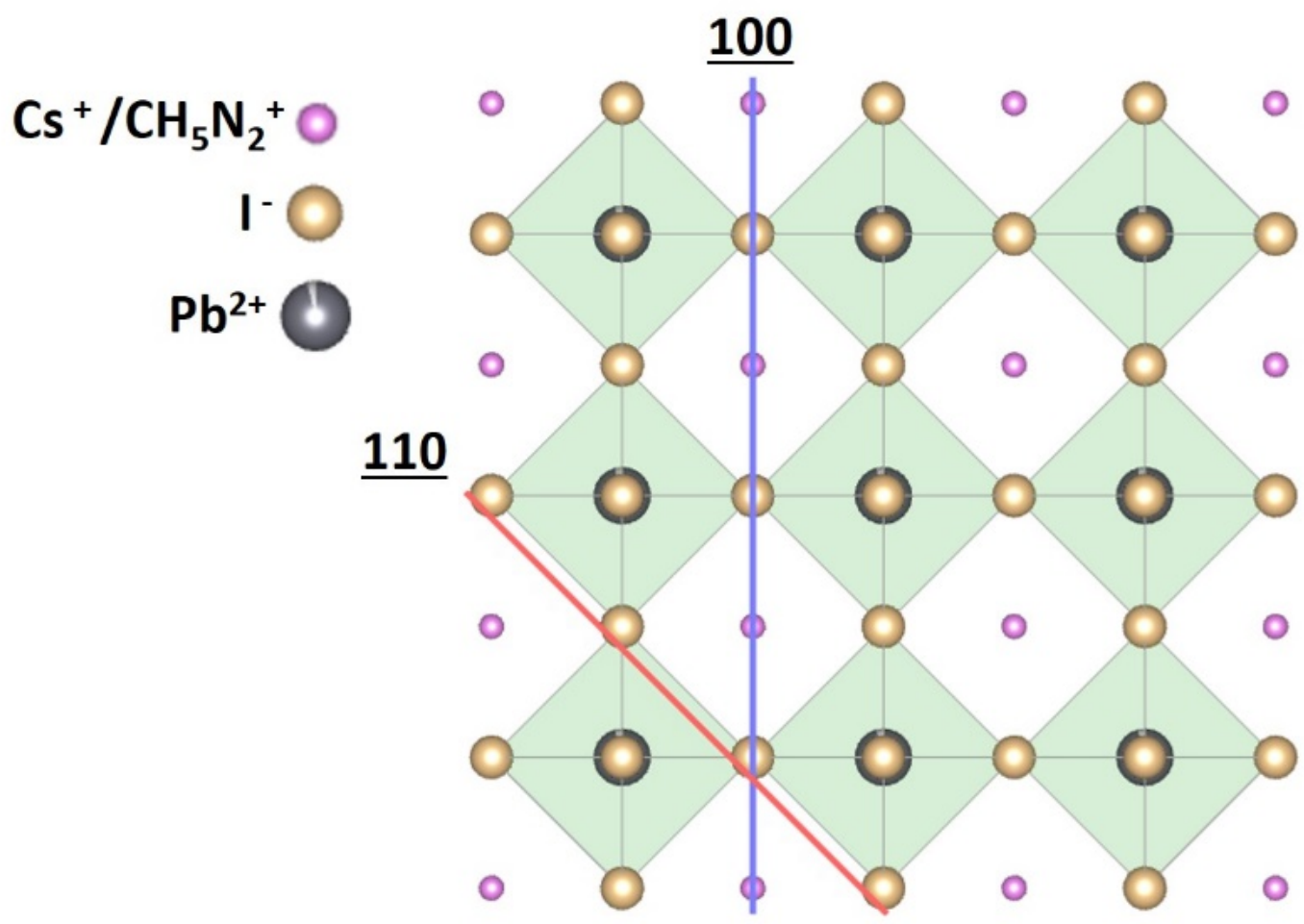

Figure S18. Preferential orientation of perovskite crystal planes in MACl-FAPbI$_3$ (blue) and CB/IPA2-2 (red) samples, respectively.
